# Thermally Activated Delayed Fluorescence: An Extended Study of Peripherally Substituted DMAC‐TRZ Analogues

**DOI:** 10.1002/chem.71352

**Published:** 2026-07-12

**Authors:** Andrea Landi, Ottavia Racchi, D. K. Andrea Phan Huu, Shohei Fukushima, Katsuaki Suzuki, Hironori Kaji, Cristina Sissa, Anna Painelli

**Affiliations:** ^1^ Department of Chemistry, Life Science and Environmental Sustainability, Parco Area delle Scienze 17/a University of Parma Parma Italy; ^2^ Department of Mathematical, Physical and Computer Sciences University of Parma Parma Italy; ^3^ Institute for Chemical Research Kyoto University Uji Kyoto Japan

**Keywords:** chemistry, fluorescence, moiety, oled, photoexcitation, photochemistry, TADF

## Abstract

A comprehensive experimental and theoretical study of a family of dyes of interest for organic light‐emitting diode (OLED) applications is presented. Specifically, we compare the behavior of structurally related dyes obtained by peripheral substitution of a prototypical thermally activated delayed fluorescence (TADF) dye, DMAC‐TRZ. Decorating the donor (DMAC) moiety with two phenyl groups, a blue‐shift of the emission is obtained, as sought, and an increase of the spin–orbit coupling, suggesting amplified TADF. Quite surprisingly, subtle geometrical rearrangements of the two peripheral groups upon photoexcitation suppress again spin–orbit coupling, leading overall to a similar photophysics as DMAC‐TRZ.

## Graphical abstract

A comprehensive experimental and theoretical study is presented of a family of TADF dyes. Comparing the behavior of structurally related dyes, we uncover the subtle and somewhat unexpected role of conformational degrees of freedom associated with peripheral groups that affect the delicate interplay of excited states that governs spin–orbit coupling.

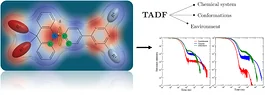

## 1 Introduction

Third‐generation organic light‐emitting diodes (OLEDs) rely on a delicate phenomenon dubbed thermally activated delayed fluorescence (TADF), where a tiny energy gap between the lowest excited singlet and triplet states ($\Delta E_{ST}$) allows the thermal conversion of electrically generated dark triplet states into bright singlets thus increasing the maximum theoretical internal quantum yield from the disappointing 25% of the first generation OLED to the promising 100%. After the original proposal by Adachi [1, 2], a wealth of research activity spurred in the field [3, 4, 5, 6, 7, 8], leading to several strategies toward optimal TADF emitters, including through bond [2, 9, 10, 11, 12, 13] and through space donor–acceptor (DA) structures [14, 15, 16], multiresonant structures [17, 18, 19, 20] and more complex strategies, like, for example, hyperfluorescent systems [21, 22, 23]. Even limiting the attention to the basic through‐bond DA structures, the physics of TADF is far from trivial: the two basic requirements for TADF, that is, a small $\Delta E_{ST}$ and a sizable spin–orbit coupling (SOC) matrix elements, are indeed mutually exclusive. The minimization of $\Delta E_{ST}$ relies in fact on the minimization of the overlap between the D and A frontier orbitals, so that the lowest excited states, with a predominant charge transfer (CT) character, $D^{+}A$ 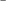, have two unpaired electrons on virtually disconnected molecular moieties, leading, according to the El‐Sayed rule [24], to vanishingly small SOC. Efficient TADF emitters then require the active involvement of local excited (LE) triplet states [4, 25, 26, 27, 28] as well as of vibronic coupling [13, 26, 29, 30, 31, 32]. The resulting scenario is made even more complex by environmental effects: the medium polarity affects in different ways the energies and the nature of CT and LE states, with a direct impact on SOC and $\Delta E_{ST}$ [6, 32, 33, 34]. Moreover, the medium rigidity affects the conformational structure and mobility of the dye then profoundly altering its photophysics [35, 36, 37, 38, 39].

Disentangling the different phenomena is a challenging task. Here, we present a detailed experimental and computational study of a series of TADF‐emitters, recently proposed by Kaji and coworkers [12, 40, 41]. Starting from a prototypical dye, DMAC‐TRZ, whose photophysics is well understood [36], we explore the effect of varying either the donor or the acceptor moiety, discussing how conformational degrees of freedom associated with peripheral groups, seemingly marginally involved in the TADF process, may largely affect the delicate TADF photophysics.

## 2 Spectroscopic Characterization

Cyclohexane (Carlo Erba, purity ≥ 99.9%), Toluene (Sigma–Aldrich, purity ≥ 99.5%) and Chloroform (Sigma–Aldrich, purity ≥ 99.8%) were utilized to prepare the solutions. UV–vis absorption spectra were recorded using a Perkin–Elmer Lambda650 spectrophotometer. Fluorescence spectra were measured employing an Horiba Jobin–Yvon Fluoromax‐3 fluorimeter. The resultant intensity profiles were corrected for the excitation intensity and the detector sensitivity. Measurements in degassed solvent were performed after three freeze–pump–thaw cycles. To avoid inner filter effects on fluorescence spectra, solutions with absorbance < 0.1 were prepared. Absorbance and fluorescence spectra were acquired using a quartz cuvette with an optical path length of 1 cm. The fluorescence quantum yield was determined versus quinine sulfate dissolved in a 0.05M $H_{2}{\mathrm{SO}}_{4}$ solution as a reference ($\Phi_{f}$ = 0.55) [42]. Lifetime decay measurements of the four systems were measured in cyclohexane, toluene, and chloroform using an FLS1000 Edinburgh Fluorimeter, upon excitation of the sample with a pulsed diode laser at 375 nm. Collected data were represented on a log–log scale, and two complementary techniques were adopted to analyze the dynamics over a 200 μs time scale. For the initial decay phase, the time‐correlated single photon counting (TCSPC) technique was utilized with a laser pulse period of 2 μs. For the intermediate time scale, the same technique was applied with an extended laser pulse period of 20 μs. To capture longer time scales, the Multichannel Scaling (MCS) technique was adopted, using a laser pulse period of 200 μs. Figure 1 shows the structures of the molecules discussed in this paper. Starting with the prototypical DMAC‐TRZ emitter, whose structure is shown in the center of the figure, four different molecules are considered, substituting the DMAC donor with 10H‐spiro[acridine‐9,9
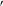
‐fluorene] (FA) or 9,9‐diphenyl‐9,10‐dihydroacridine (PA) and substituting the TRZ acceptor with 2,4‐di‐(adamantan‐1‐yl)‐6‐phenyl‐1,3,5‐triazine (TA) and 2‐phenyl‐1,3,5‐triazine (TH). DMAC‐TRZ was acquired from Merck, FA‐TRZ [43], PA‐TRZ [43], and DMAC‐TA [12] were prepared according to previously reported procedures. DMAC‐TH was prepared following the procedure described in the Supporting Information.

**FIGURE 1:**
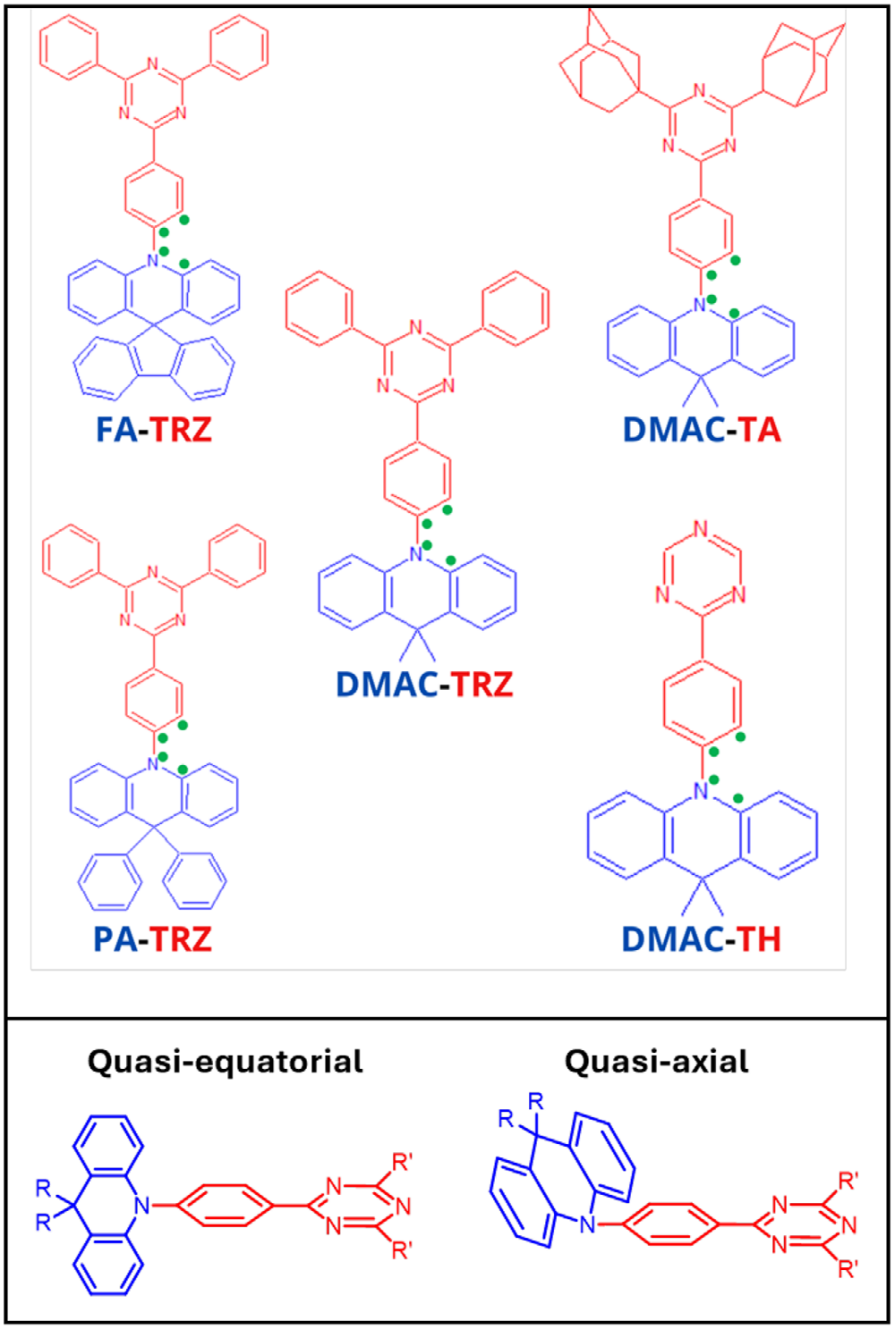
Top: The chemical structures of the molecules discussed in this work. The green dots mark the four atoms that in each molecule define the dihedral angle of interest (δ). Bottom: A sketch of the two limiting molecular conformations: The quasi‐equatorial (QE) conformation where the two molecular fragments are orthogonally arranged (δ∼90), and the quasi‐axial conformation, with $\delta ∼0^{\circ }$ but, because of sterical hindrance, a bent structure. Specific geometrical arrangements of the different dyes are shown in Figure S11.

Figure 2 collects absorption and fluorescence spectra of FA‐TRZ, PA‐TRZ, DMAC‐TA, and DMAC‐TH dissolved in cyclohexane, toluene, and chloroform (spectra of DMAC‐TRZ can be found in ref. [44]). Main spectroscopic features in toluene are listed in Table 1. Steady‐state spectra of the four dyes are similar to those of the parent DMAC‐TRZ molecule. All dyes show a non‐solvatochromic absorption band and a strongly solvatochromic emission. DMAC‐TH is a much smaller molecule than all other systems: the smaller cavity dimension explains the larger emission solvatochromism observed for this dye when compared to the other species.

**FIGURE 2:**
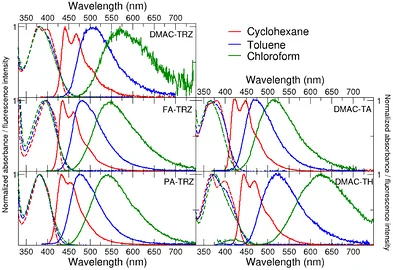
Normalized absorbance and fluorescence spectra (dashed and continuous lines, respectively) of the five molecules of interest shown in Figure 1, collected in cyclohexane (red), toluene (blue), and chloroform (green).

**TABLE 1:** Spectroscopic data in toluene.

Molecule	$\lambda_{\max}^{\mathrm{abs}}$ (nm)	$\lambda_{\max}^{\mathrm{em}}$ (nm)	ϕ (air)	ϕ (degassed)
FA‐TRZ	397	484	0.21	0.53
PA‐TRZ	382	477	0.21	0.47
DMAC‐TRZ	382	510	0.22^a^	0.67^a^
DMAC‐TA	368	473	0.19	0.34
DMAC‐TH	372	525	0.12	0.31

^a^ From ref.  [45].

The most significant deviation from the standard (DMAC‐TRZ) behavior is observed for DMAC‐TH whose absorption spectrum in cyclohexane shows, apart from the main peak (at ∼368 nm) an additional feature at 397 nm (this feature is less prominent in toluene and a minor feature is also observed in DMAC‐TA in cyclohexane). Detailed analysis, relying on fluorescence excitation spectra measurements (see Figure S8) allows us to safely ascribe this feature to the presence of two conformers of DMAC‐TH in non‐polar solvents.

## 3 Computational Study

DFT (M062X/6‐31g(d)) calculations were performed using Gaussian 16 package [46], both in gas phase and in implicit solvent (PCM) for the five molecules of interest to address the ground state geometry. The most relevant geometrical parameter is the dihedral angle (δ) between D and A groups as illustrated in Figure 1. Therefore, we calculated the ground state energy of the molecules for different values of the dihedral angle, while allowing all other coordinates to fully relax. Relevant results (Figure S9) show that for all molecules, much as for DMAC‐TRZ [47], the lowest energy minimum corresponds to the orthogonal or quasi‐equatorial geometry, but that, with the only exception of FA‐TRZ, having a bulky and rigid D‐group, a quasi‐axial conformation, where the donor and acceptor units are periplanar (Figure S11), is also possible, with marginal variations due to the solvent polarity. However, clear experimental evidence of the presence of the two conformers is found only for DMAC‐TH in non‐polar solvents.

To address excited states, we resort to TD‐DFT (M06‐2X/6‐31g(d), Tamm‐Dancoff approximation). The choice of the functional and basis set is in line with previous work on DMAC‐TRZ [36, 44]. More in detail, Tables S1 and S2 compare results obtained for two dyes with different basis sets, showing a marginal dependence of low energy excitation frequencies, in line with results from extensive benchmark in ref. [48]. As for excited states, we limit attention to gas‐phase results, in view of the limitations of current implementations of implicit solvent models that are particularly serious when addressing excited states [6, 49, 50]. Table 2 reports the vertical energies of the first five excited states (one singlet and four triplets) calculated at the ground state equilibrium geometry. Figure 3 shows the vertical energies from the ground state equilibrium geometry and the absolute value of the SOC matrix element between the lowest excited singlet and triplet states calculated (Orca 4.1 package [51]) as a function of the dihedral angle δ via rigid scans (SOC data from relaxed scans are shown in Figure S12. Figure S13 compares the SOC calculated for PA‐TRZ and DMAC‐TRZ.).

**TABLE 2:** Gas phase vertical energies (eV) of the five lowest excited states of FA‐TRZ, PA‐TRZ, DMAC‐TRZ, DMAC‐TA, and DMAC‐TH at their ground state minimum energy geometry.

	*T* 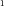	*S* 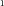	*T* 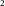	*T* 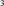	*T* 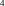
FA‐TRZ	3.50(CT)	3.51(CT)	$3.75\;({\mathrm{LE}}_{A})$	$3.78\;({\mathrm{LE}}_{D})$	$3.85\;({\mathrm{LE}}_{D})$
PA‐TRZ	3.69(CT)	3.71(CT)	$3.75\;({\mathrm{LE}}_{A})$	$3.97\;({\mathrm{LE}}_{D})$	$3.99\;({\mathrm{LE}}_{A})$
DMAC‐TRZ	3.44(CT)	3.45(CT)	$3.78\;({\mathrm{LE}}_{A})$	$3.83\;({\mathrm{LE}}_{D})$	$3.97\;({\mathrm{LE}}_{A})$
DMAC‐TA	3.51(CT)	3.52(CT)	$3.83\;({\mathrm{LE}}_{A})$	$3.86\;({\mathrm{LE}}_{D})$	$4.03\;({\mathrm{LE}}_{D})$
DMAC‐TH	3.36(CT)	3.36(CT)	$3.83\;({\mathrm{LE}}_{A})$	$3.83\;({\mathrm{LE}}_{D})$	$4.04\;({\mathrm{LE}}_{D})$

*Note*: The primary contribution concerning the nature of the states is specified in parentheses.

**FIGURE 3:**
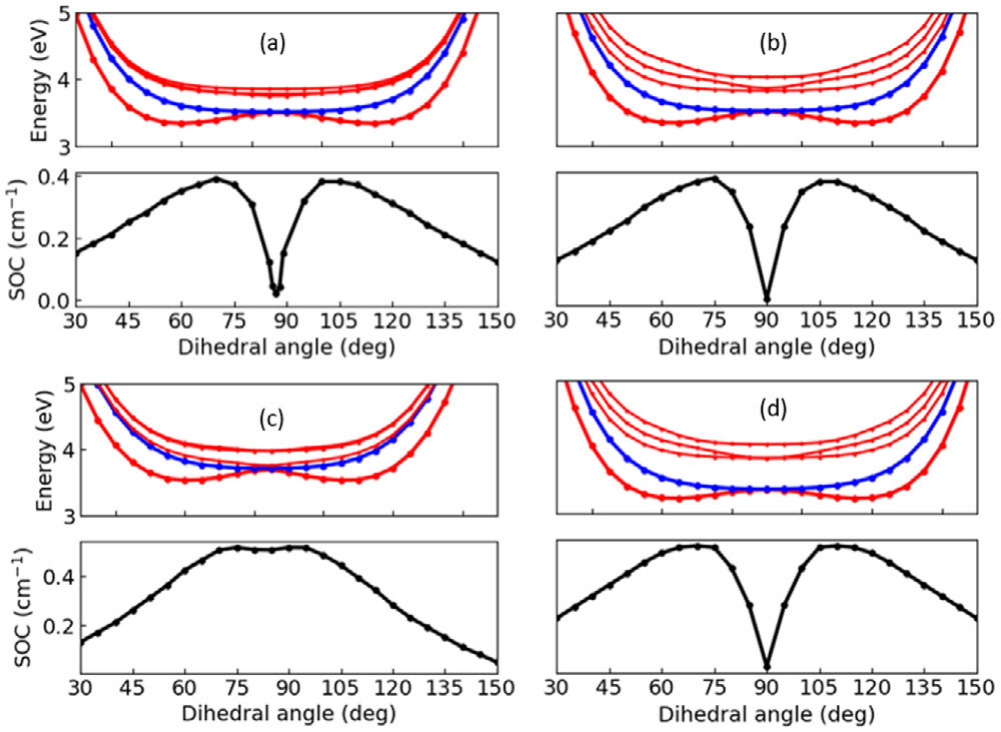
The four quadrants show the results of rigid scan calculations for (a) FA‐TRZ, (b) DMAC‐TA, (c) PA‐TRZ, and (d) DMAC‐TH. For each molecule, the bottom panel shows the $|\left\langle S_{1}\right|H_{SOC}|T_{1}\rangle |$ and the top panel shows vertical transition energy for the lowest singlet state ($S_{1}$, blue trace) and for the four lowest triplet states (red lines).

All molecules behave essentially as DMAC‐TRZ [44], with vanishing (or almost so) $|\left\langle S_{1}\right|H_{SOC}|T_{1}\rangle |$ at the equilibrium geometry ($∼90^{\circ }$), with the notable exception of PA‐TRZ, which shows a sizable spin orbit coupling at the equilibrium geometry. The reason for this anomaly lies in close proximity of the CT triplet ($T_{1}$) with a localized triplet ($T_{2}$), as shown in Table 2. Accordingly, the two states are largely mixed, leading to enhanced SOC as well as to an increased $\Delta E_{ST}$. Since both properties largely affect TADF, in the next section we present a detailed analysis of the time‐resolved fluorescence of the PA‐TRZ in liquid solvents. For comparison purposes, we also address DMAC‐TRZ.

Before proceeding, we notice that at the equilibrium QE geometry we calculate for all molecules negligible oscillator strength to the $S_{1}$ state (see Table S3) and, with the exception of PA‐TRZ, negligible SOC. In these conditions, one would not expect any sizable absorption/fluorescence intensity from the $S_{1}$ state, nor any TADF. However, as discussed in refs. [6, 36, 44], the fairly flat minimum around the equilibrium geometry leads to a finite thermal population of states with a dihedral angle deviating from orthogonality. The observed absorption/fluorescence intensity and the sizable ISC/RISC rates are precisely due to this thermal population (see also Section S6).

## 4 TADF in Solution

Figure 4 shows the time evolution of the luminescence intensity measured for DMAC‐TRZ and PA‐TRZ dissolved in cyclohexane, toluene, and chloroform. Data are analyzed following the procedure described in ref. [52] (see Supporting Information) to extract the rate constants reported in Table 3. The estimated rate constants of DMAC‐TRZ are in line with the results reported in ref. [36]. Unfortunately, the rate constants evaluated for PA‐TRZ in cyclohexane are affected by a large uncertainty, due to the weak experimental TADF response, which makes the delayed fluorescence component very noisy. However, the most notable result is that the rate constants of PA‐TRZ are of the same order of magnitude as those of DMAC‐TRZ. This result sharply contrasts with the results in Figure 3, PA‐TRZ has indeed a large SOC at the ground state equilibrium geometry and the tiny energy gap between $T_{2}$ and $T_{1}$ would also suggest a direct involvement of $T_{2}$ to $S_{1}$ transfer, suggesting efficient TADF.

**FIGURE 4:**
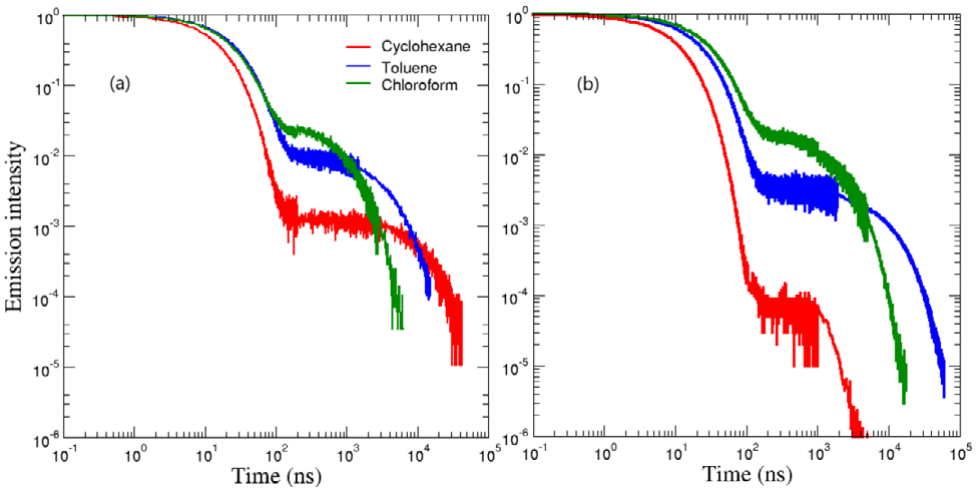
Time evolution of the integrated luminescence measured for DMAC‐TRZ (a) and PA‐TRZ (b) in three different solvents.

**TABLE 3:** Experimental ISC and RISC rate constants (in $s^{-1}$) for DMAC‐TRZ and PA‐TRZ in cyclohexane, toluene, and chloroform.

	Cyclohexane	Toluene	Chloroform
DMAC‐TRZ			
$k_{\mathrm{ISC}}$	$3.0-5.5\cdot 10^{7}$	$1.6-3.5\cdot 10^{7}$	$2.2-3.9\cdot 10^{7}$
$k_{\mathrm{RISC}}$	$0.9-1.8\cdot 10^{5}$	$2.4-5.1\cdot 10^{5}$	$1.3-2.3\cdot 10^{6}$

PA‐TRZ			
$k_{\mathrm{ISC}}$	$0.09-4.7\cdot 10^{7}$	$2.8-4.3\cdot 10^{7}$	$2.0-3.4\cdot 10^{7}$
$k_{\mathrm{RISC}}$	$0.01-1.1\cdot 10^{6}$	$1.8-2.7\cdot 10^{5}$	$0.85-1.2\cdot 10^{5}$

This inconsistency can be traced back to the flexibility introduced by the phenyl rings in the PA moiety. As stated above, in the ground state geometry the $S_{1}$ and $T_{1}$ states of PA‐TRZ (with main CT character, see Table 2) move slightly to the blue, going very close to the local excited triplet states. The resulting mixing explains the large SOC observed in the orthogonal configuration corresponding to the ground state geometry of PA‐TRZ. However, ISC and rISC occur from the $S_{1}$ and $T_{1}$ states, respectively. Defining the geometry for $T_{1}$ of PA‐TRZ is non‐trivial, since different minima with similar absolute energies are reached depending on the starting geometry, but Figure S14 shows that the $S_{1}$ relaxed geometry is well‐defined. Specifically, as for the main dihedral angle, much as it happens for DMAC‐TRZ, it remains orthogonal (Figure S14). At variance with DMAC‐TRZ, in PA‐TRZ other degrees of freedom enter into play. As shown in Figure 5e,f, in the optimized $S_{1}$ geometry, a rearrangement of the phenyl groups is accompanied by a planarization of the conjugated donor core with respect to the $S_{0}$ geometry (Figure 5c,d). When going from the $S_{0}$ to the $S_{1}$ geometry, the deviation of the N atoms from the donor plane (defined in [53]) is reduced, indicating increased donor conjugation in the excited state. This geometric reorganization leads to a completely different distribution of the triplet state energies, as shown in Figure 5. Concomitantly, the SOC matrix element at the $S_{1}$ equilibrium geometry drops down to 0.05 ${\mathrm{cm}}^{-1}$, leading to a situation that strongly resembles what is observed for DMAC‐TRZ and the other dyes discussed in this paper.

**FIGURE 5:**
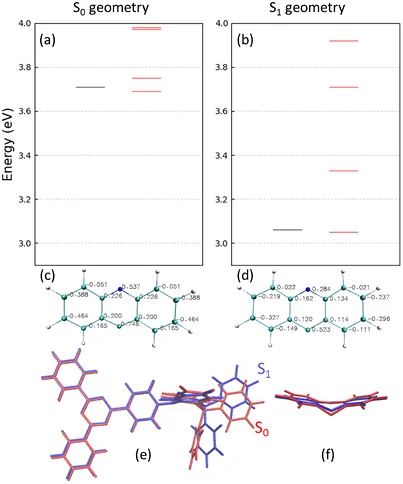
Panels (a) and (b) show the absolute energies of $S_{1}$ (black) and of the first few triplet states (red) calculated for PA‐TRZ at the $S_{0}$ and $S_{1}$ equilibrium geometries, respectively. Panels (c) and (d) show the geometry of the donor fragment of PA‐TRZ at the $S_{0}$ and $S_{1}$ geometries, respectively. The labels on each atom show the deviation from planarity (in Angström), defined as in Ref.  [53] accounting only for non‐hydrogen atoms. Panel (f) shows a superposition of $S_{0}$ and $S_{1}$ equilibrium geometries. Panel (e) is the same as panel (f), but for the molecular fraction corresponding to the donor core in panel (c) and (d).

For a more detailed analysis, Figure 6 shows the results of rigid scan calculation on PA‐TRZ and DMAC‐TRZ obtained for the $S_{1}$ geometry (for comparison, Figure S14 shows the results obtained for a relaxed scan). The difference with the rigid geometry scan relevant to the ground state (Figure 3c) is impressive and brings the PA‐TRZ system more in line with DMAC‐TRZ. As discussed above, the qualitatively different results obtained for the SOC matrix element in a dihedral angle scan run optimizing $S_{0}$ or $S_{1}$ geometry, as observed in PA‐TRZ, is associated with the additional degrees of freedom introduced by the two phenyl groups. Quite interestingly, for DMAC‐TRZ results obtained from rigid scans around the $S_{0}$ geometry in ref. [44] or $S_{1}$ geometries in Figure 6 are similar, confirming again the highly non‐trivial role of the phenyl substituents.

**FIGURE 6:**
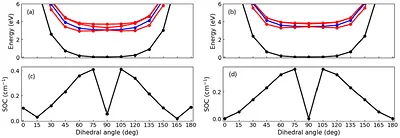
(a,b) Energies of the ground state (black), the $S_{1}$ state (blue) and the first few triplet states (red) calculated as a function of the dihedral angle for the $S_{1}$ optimized geometry (rigid scan) PA‐TRZ and DMAC‐TRZ, respectively.(c,d) The corresponding SOC matrix element between $S_{1}$ and $T_{1}$ PA‐TRZ and DMAC‐TRZ, respectively.

To simulate this complex behavior, we adapted the model originally proposed and successfully applied to DMAC‐TRZ. A detailed derivation of the model can be found in Supporting Information, here we just outline the main ingredients. As for DMAC‐TRZ, we account for four diabatic (basis) states to describe the low‐energy physics of the system: two singlet states, corresponding to the neutral DA and the charge‐transfer (CT) $D^{+}A$ 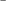 states, and two triplet states, the CT triplet and an effective localized triplet. The model accounts for the mixing between the two singlet states and between the two triplet states, with a mixing matrix element that varies with the dihedral angle. Interestingly, in DMAC‐TRZ the two matrix elements that mix the two singlets and the two triplets vanish in the orthogonal geometry, but in PA‐TRZ, to describe the sizable SOC calculated in this geometry, we also introduce a finite coupling between the two triplets in this geometry. Finally, SOC matrix elements are introduced by mixing the neutral singlet with the CT triplet and the CT singlet with the localized triplet. All model parameters are fixed to reproduce TD‐DFT results. Much as in DMAC‐TRZ, a molecular vibrational mode is introduced accounting for the geometrical relaxation when going from neutral to CT state, but in the case of PA‐TRZ, in order to account for the additional flexibility introduced by the phenyl rings, we also allow for the relaxation of the local excited triplet along the same coordinate. Finally, much as in DMAC‐TRZ, a solvation coordinate accounts for environmental effects, relying on the Onsager model. Calculated ISC and RISC rate constants are shown in Table 4, and a comparison between experimental and calculated RISC rate constants is shown in Figure 7a for DMAC‐TRZ and Figure 7b for PA‐TRZ.

**TABLE 4:** Computed ISC and RISC rate constants (in $s^{-1}$) for DMAC‐TRZ and PA‐TRZ in cyclohexane, toluene, and chloroform.

	Cyclohexane	Toluene	Chloroform
DMAC‐TRZ			
$k_{\mathrm{ISC}}$	$4.2\times 10^{5}$	$1.8\times 10^{6}$	$1.1\times 10^{7}$
$k_{\mathrm{RISC}}$	$4.9\times 10^{4}$	$3.1\times 10^{5}$	$2.2\times 10^{6}$

PA‐TRZ			
$k_{\mathrm{ISC}}$	$4.3\times 10^{6}$	$1.0\times 10^{6}$	$3.7\times 10^{6}$
$k_{\mathrm{RISC}}$	$2.8\times 10^{5}$	$9.0\times 10^{4}$	$6.2\times 10^{5}$

**FIGURE 7:**
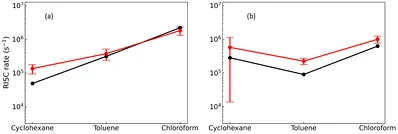
Comparison between calculated (black) and experimental (red) RISC rate constants for DMAC‐TRZ (a) and PA‐TRZ (b) in different solvents.

For both compounds, the model satisfactorily reproduces experimental results, confirming that PA‐TRZ rate constants are similar to those of DMAC‐TRZ. In both cases, calculated ISC rates are largely underestimated, a problem that can be ascribed to the presence of several triplet states (not accounted for in the proposed mode) below the lowest excited singlet offering effective decay channels.

## 5 Discussion and Conclusions

TADF is an intriguing but delicate phenomenon that requires a precise energetic alignment of the lowest singlet and triplet states and sizable spin–orbit coupling. In donor–acceptor TADF systems, the energy alignment is typically reached by breaking the D–A conjugation so that the lowest singlet and triplet states have an almost pure CT character. A moderate degree of conjugation, facilitated by conformational flexibility, is required to generate a non‐zero oscillator strength, enabling the emission and supporting the mixing of charge‐transfer and locally excited triplet states, while also ensuring a finite spin orbit coupling. Controlling all these interactions that are in turn largely affected by environmental degrees of freedom, is a challenging task.

In this work, relying on the detailed understanding reached on the prototypical TADF dye, DMAC‐TRZ, we undertook a thorough experimental and theoretical analysis of four related dyes. Out of them, PA‐TRZ was singled out for a detailed analysis due to a comparatively large SOC calculated for this system. However, in spite of the large SOC, PA‐TRZ has a similar TADF behavior as DMAC‐TRZ. This anomaly can be ascribed to the more flexible structure of PA‐TRZ. In PA‐TRZ two phenyl groups replace the two methyl groups of the DMAC unit. This apparently innocent substitution, meant essentially to blue‐shift the emission band, actually brings a local triplet state very close to the CT triplet, then increasing the CT‐LE mixing and explaining the large SOC calculated at the ground state equilibrium geometry. However, in the excited state $S_{1}$ geometry, these flexible units rearrange considerably, effectively decoupling the local excited triplets from the CT triplet and bringing back SOC to a tiny number. The lesson to be learned here is that TADF is a tricky phenomenon where many interactions must be controlled and accounted for: among them, the conformational degrees of freedom of peripheral groups that are marginally involved in the main CT process, but that may considerably alter the delicate interplay between LE and CT triplets that guarantees sizable SOC and hence allows for TADF.

## Conflicts of Interest

The authors declare no conflicts of interest.

## Supporting information


**Supporting File 1**: chem71352‐sup‐0001‐SuppMat.pdf

## Data Availability

The input geometries for ab initio calculations and the codes for fitting and calculation of photophysical rate constants can be found at https://doi.org/10.5281/zenodo.19206020.
