# Supplementary material for: Thermally Activated Delayed Fluorescence: An Extended Study of Peripherally Substituted DMAC‐TRZ Analogues
Source: Chemistry. 2026 Jul 12;32(39):e71352. doi: 10.1002/chem.71352 (PMC13715487; doi:10.1002/chem.71352)
Supplement: Supplementary file 1 [file CHEM-32-e71352-s001.pdf]

Thermally activated delayed fluorescence: the subtle role of side groups†

Andrea Landi,[a,‡] Ottavia Racchi,[a,‡] D. K. Andrea Phan Huu,[a,b,*] Shohei Fukushima,[c] Katsuaki Suzuki,[c] Hironori Kaji,[c] Cristina Sissa,[a] Anna Painelli[a]

1 Materials

1.1 General

All reagents were purchased from commercial sources and used without further purification. All reactions were carried out under an argon atmosphere. ^{1}H and ^{13}C NMR spectra were recorded with a JEOL ECA-600 spectrometer ($600\,\mathrm{MHz}$ for ^{1}H, $151\,\mathrm{MHz}$ for ^{13}C). Chemical shifts are reported in δ (ppm) using residual protons in the deuterated solvents for ^{1}H NMR and using solvent peaks for ^{13}C NMR as internal standards. Thin layer chromatography (TLC) was performed on plates coated with $0.25\,\mathrm{mm}$ of silica gel 60F-254 (Merck). Column chromatography was performed using PSQ 60B silica (Fuji Silysia). DMAC-TRZ was acquired from Merk, FA-TRZ[1], PA-TRZ[1], and DMAC-TA[2] were synthesized according to previously reported procedures, while DMAC-TH was synthesized as described below.

1.2 Synthesis of DMAC-BH

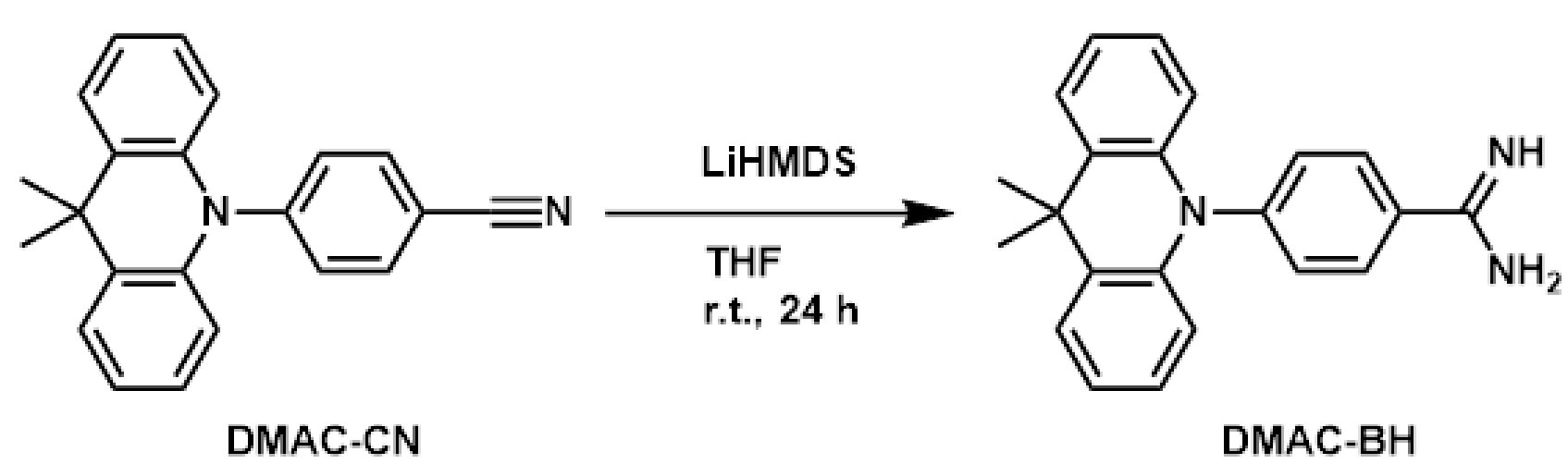

Figure S1 Synthesis of DMAC-BH.

To a stirred solution of DMAC-CN[3] (142 mg, 0.47 mmol) in dry THF (2.0 mL) at 0 °C was added dropwise LiHMDS in THF (1.3 M, 0.5 mL, 0.65 mmol). The mixture was stirred for 1 h at 0 °C. After stirring for 24 h at room temperature, the mixture was acidified to pH 3 with 1 M HCl (aq), and the resulting precipitate was collected. Then, the solid was suspended in water and basified to pH 12 with 2 M NaOH (aq). The resulting precipitate was filtered and collected to give 115 mg (0.35 mmol) of DMAC-BH as a white solid in 74% yield, which was used without further purification.
^{1}H NMR (600 MHz, $CDCl_3$): ^{1}H NMR (600 MHz, DMSO-d6): δ 8.10 (d, J = 8.4 Hz, 2H), 7.47 (dd, J = 1.3 Hz, 7.8 Hz, 2H), 7.42 (d, J = 9 Hz, 2H), 7.03–6.90 (m, 4H), 7.42 (d, J = 7.8 Hz, 2H), 1.69 (s, 6H); ^{13}C NMR (151 MHz, DMSO-d6): δ 143.8, 140.5, 135.6, 134.8, 131.6, 130.4, 128.8, 126.4, 125.4, 121.0, 114.1, 36.03, 31.18.

[a] Department of Chemistry, Life Science and Environmental Sustainability, Parco Area delle Scienze 17/a, University of Parma, 43124 Parma, Italy.
[b] Department of Mathematical, Physical and Computer Sciences, University of Parma, Parma 43124, Italy.
[c] Institute for Chemical Research, Kyoto University, Uji, Kyoto 611-0011, Japan.
‡ These authors contributed equally to this work.

1.3 Synthesis of DMAC-TH

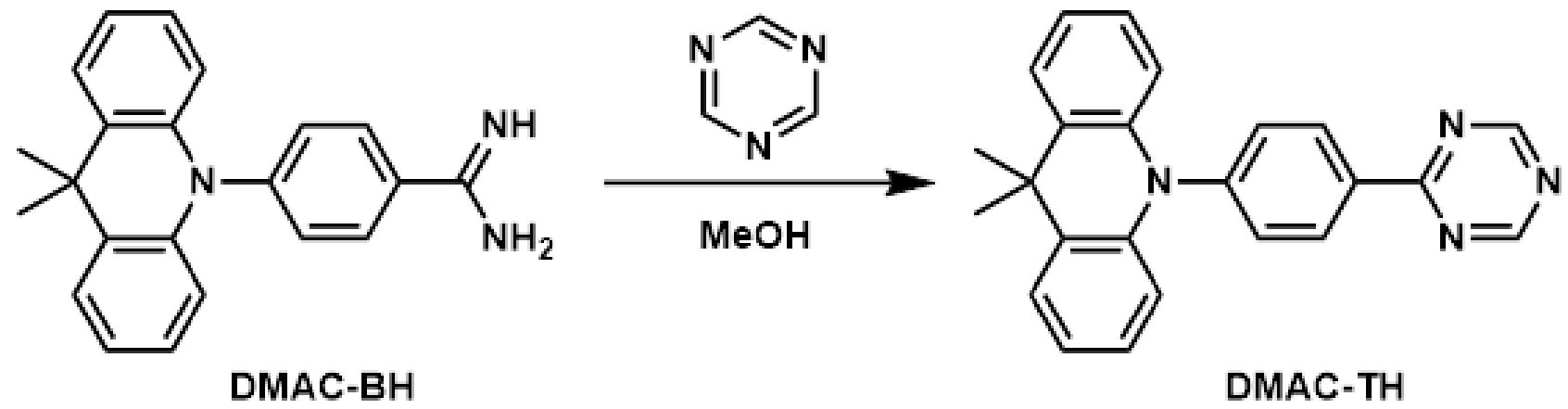

Figure S2 Synthesis of DMAC-TH.

DMAC-BH (115 mg, 0.35 mmol) and triazine (31.3 mg, 0.39 mmol) were dissolved in dry MeOH (2.0 mL) and stirred for 17h at room temperature. To the mixture, water was added and resulting precipitates were filtered and recrystallized from $CHCl_3$/Hexane. The product was further purified by silica-gel column chromatography ($CHCl_3$: hexane = 3 : 1, Rf = 0.2) to give 30.6 mg (10.1 mmol) of DMAC-TH as a colorless solid in 24% yield. ^{1}H NMR (600 MHz, DMSO-d6): δ 9.28 (s, 2H), 8.78 (d, J = 7.8 Hz, 2H), 7.53 (d, J = 8.4 Hz, 2H), 7.48 (d,d J = 1.4, 7.2 Hz, 2H), 7.01–6.93 (m, 4H), 6.34 (dd J = 1.3, 7.4 Hz, 2H), 1.70 (s, 6H); ^{13}C NMR (151 MHz, DMSO-d6): δ 170.6, 166.5, 164.1, 140.5, 134.5, 131.52, 131.49, 130.6, 126.4, 125.3, 121.0, 114.3, 36.08, 31.14; HESI-HRMS (m/z): [M+H]+ calcd. for C24H21N4, 365.17607; found, 365.17609.

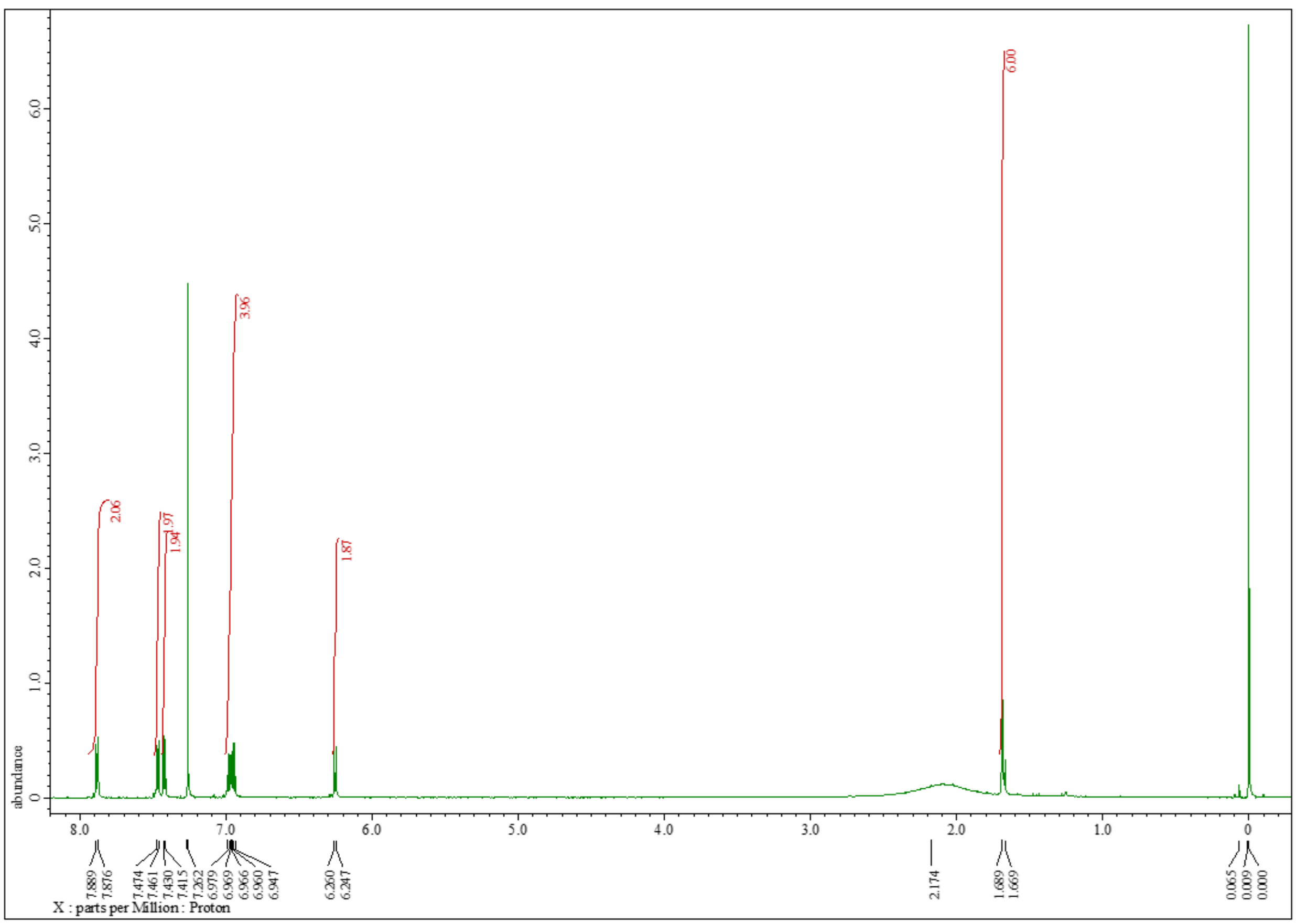

Figure S3 Solution-state ^{1}H NMR spectrum of DMAC-BH.

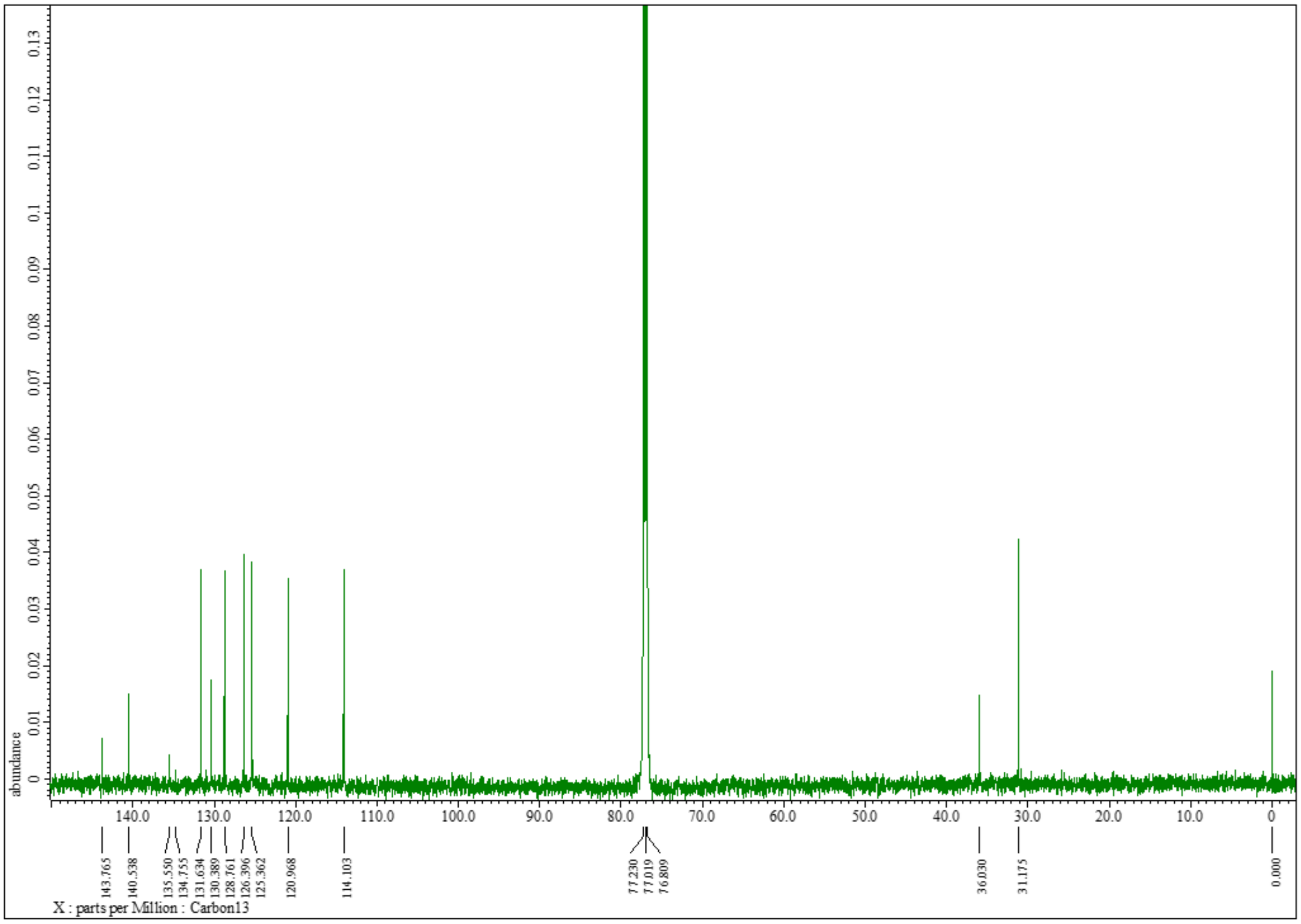

Figure S4 Solution-state ^{13}C NMR spectrum of DMAC-BH.

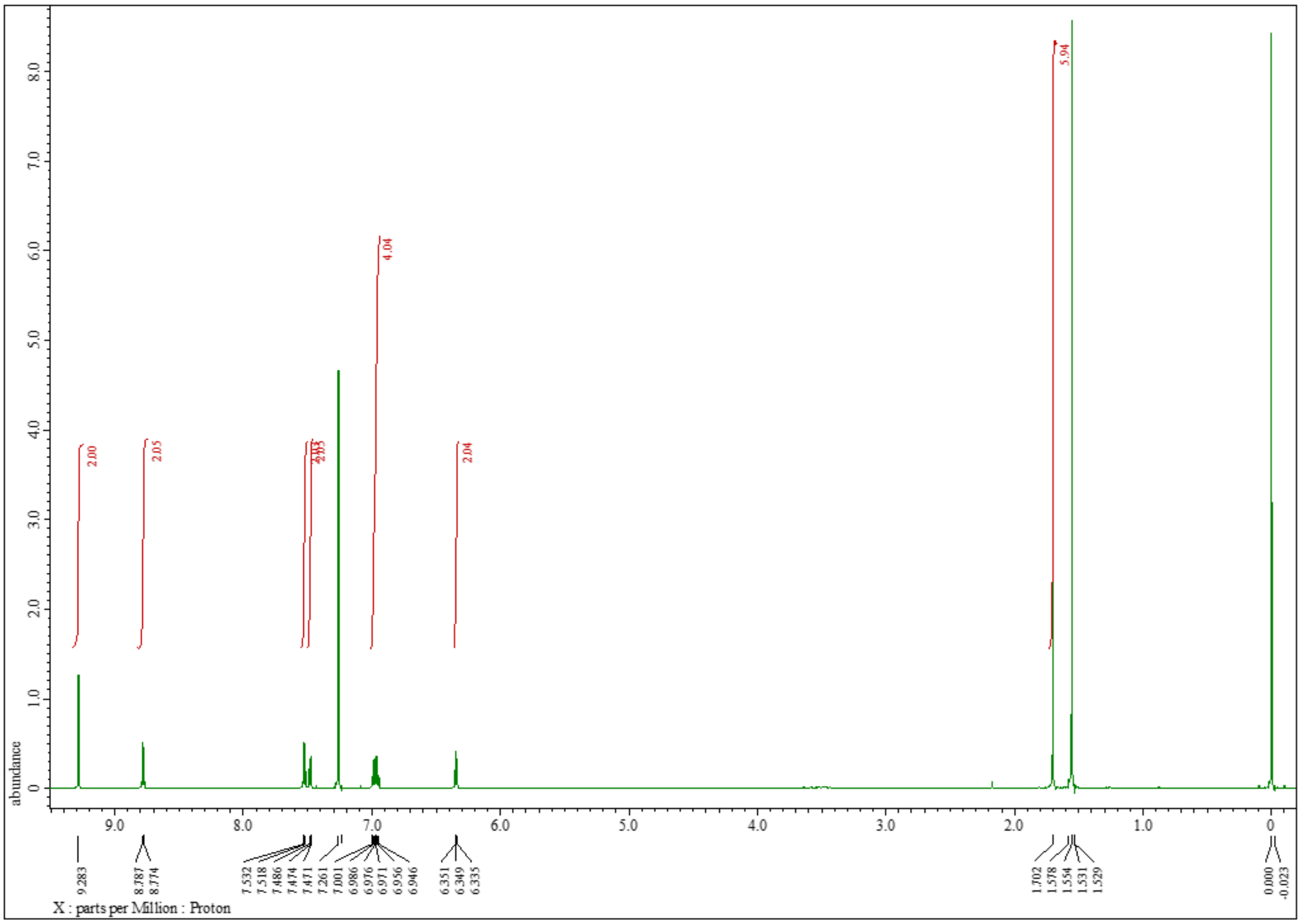

Figure S5 Solution-state ^1H NMR spectrum of DMAC-TH.

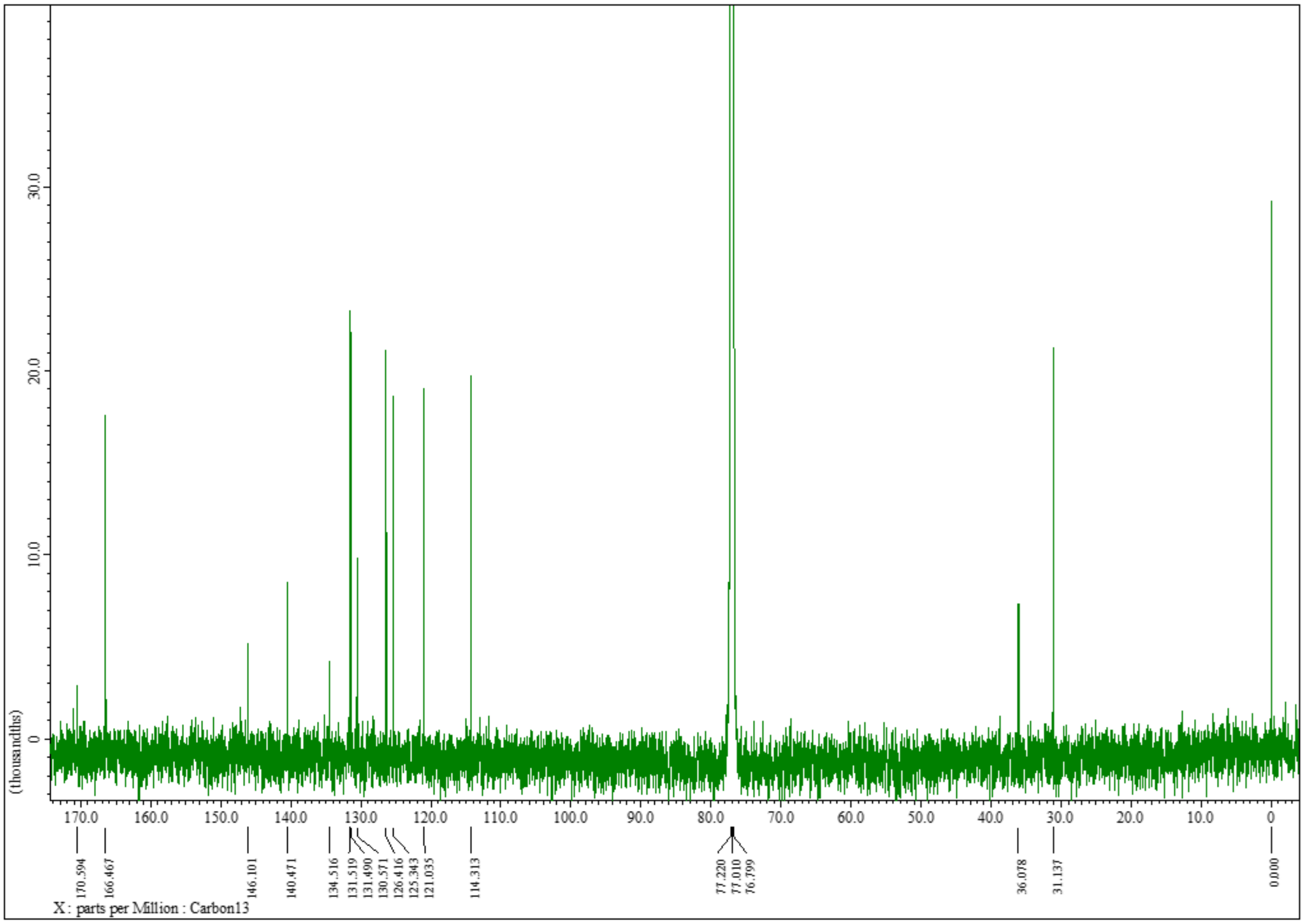

Figure S6 Solution-state ^{13}C NMR spectrum of DMAC-TH.

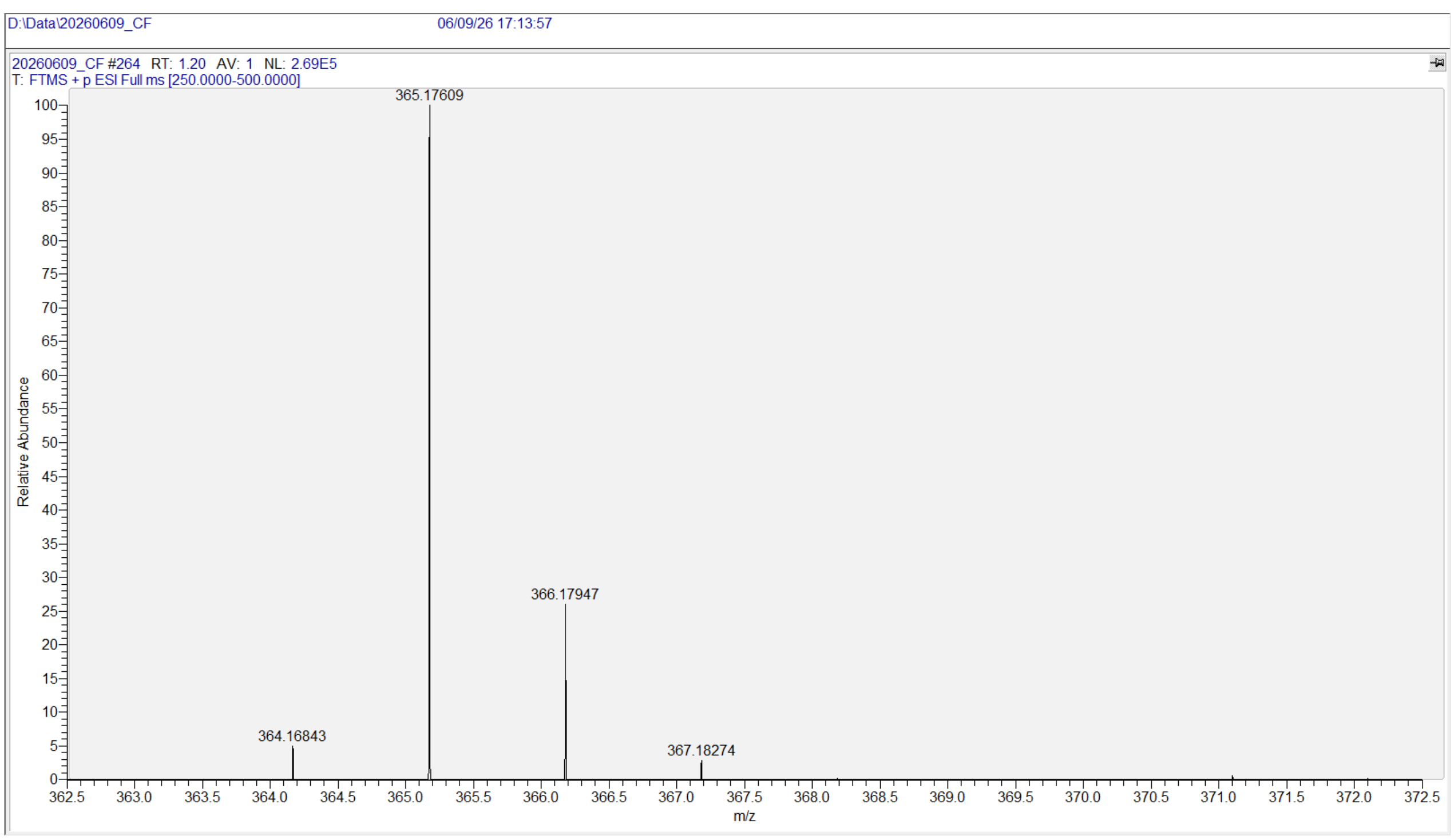

Figure S7 HESI-HRMS spectrum of DMAC-TH.

2 DMAC-TH: spectra collected at low temperature

Emission and excitation spectra were measured using an FLS1000 Edinburgh Fluorimeter equipped with a 450 W Xenon Lamp and a PMT-900 Detector, covering wavelengths from 200 to 870 nm. The solutions, prepared with 2-methyltetrahydrofuran (Sigma Aldrich, purity $\geq$ 99.5%), were cooled using an Oxford Instruments OptistatDN cryostat, which provided a controlled low-temperature exchange gas environment for the samples held in cryogenic quartz cuvettes. The 2Me-THF solutions were rapidly cooled to 77K to obtain a transparent glass.

Emission spectra (black line in Fig. S8) show two well separated peaks. Excitation spectra, collected at the wavelength corresponding to the maxima of the two emission peaks (395 and 490 nm) are distinctively different, demonstrating the presence of two different species in solution and hence supporting the hypothesis of the presence of two conformers in the sample.

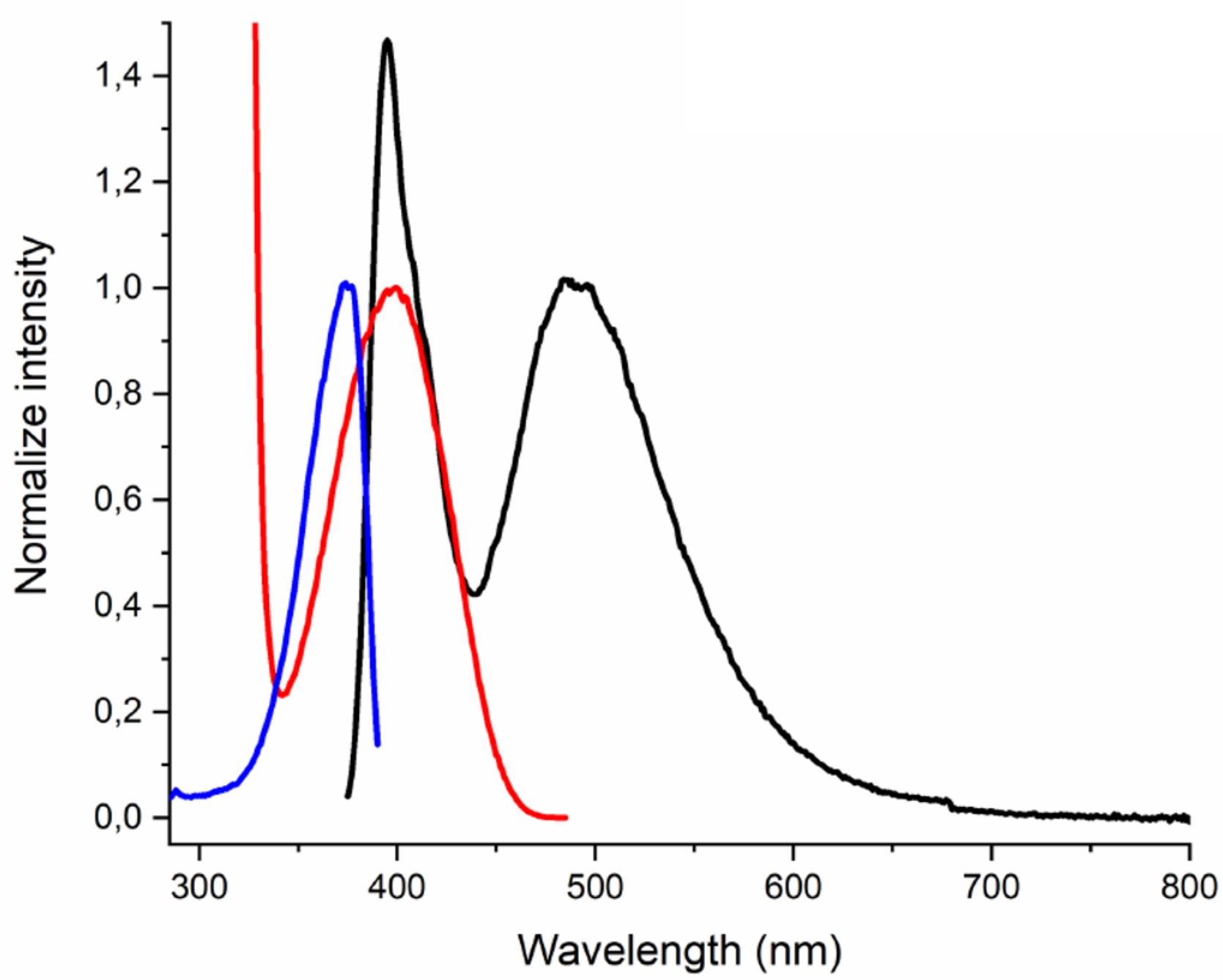

Figure S8 Emission and excitation spectra of DMAC-TH in 2MeTHF at 77K. Black: emission spectrum; Red: excitation spectrum detected at 490nm; Blue: excitation spectrum detected at 395nm

3 Ground state energy scans

Fig. S9 shows the relaxed energy scans of the ground state of the five molecules of interest. The blue dashed line defines the thermal energy at room temperature. For each system, the distribution of the possible geometries includes the quasi-equatorial ($\delta = 90°$) structure, while for DMAC-TH, DMAC-TRZ, DMAC-TA and PA-TRZ, a quasi-planar ($\delta = 0°$) structure is also possible. The optimizations performed in presence of solvent suggest that the solvation does not affect the geometries of the studied systems. As visible from Fig. S9, the quasi-axial conformation of FA-TRZ results much higher in energy with respect to the quasi-equatorial conformation. This behavior is probably due to the hindering of the fluorene unit, as shown in Fig. S10, which compares the quasi-axial conformation of DMAC-TRZ and FA-TRZ.

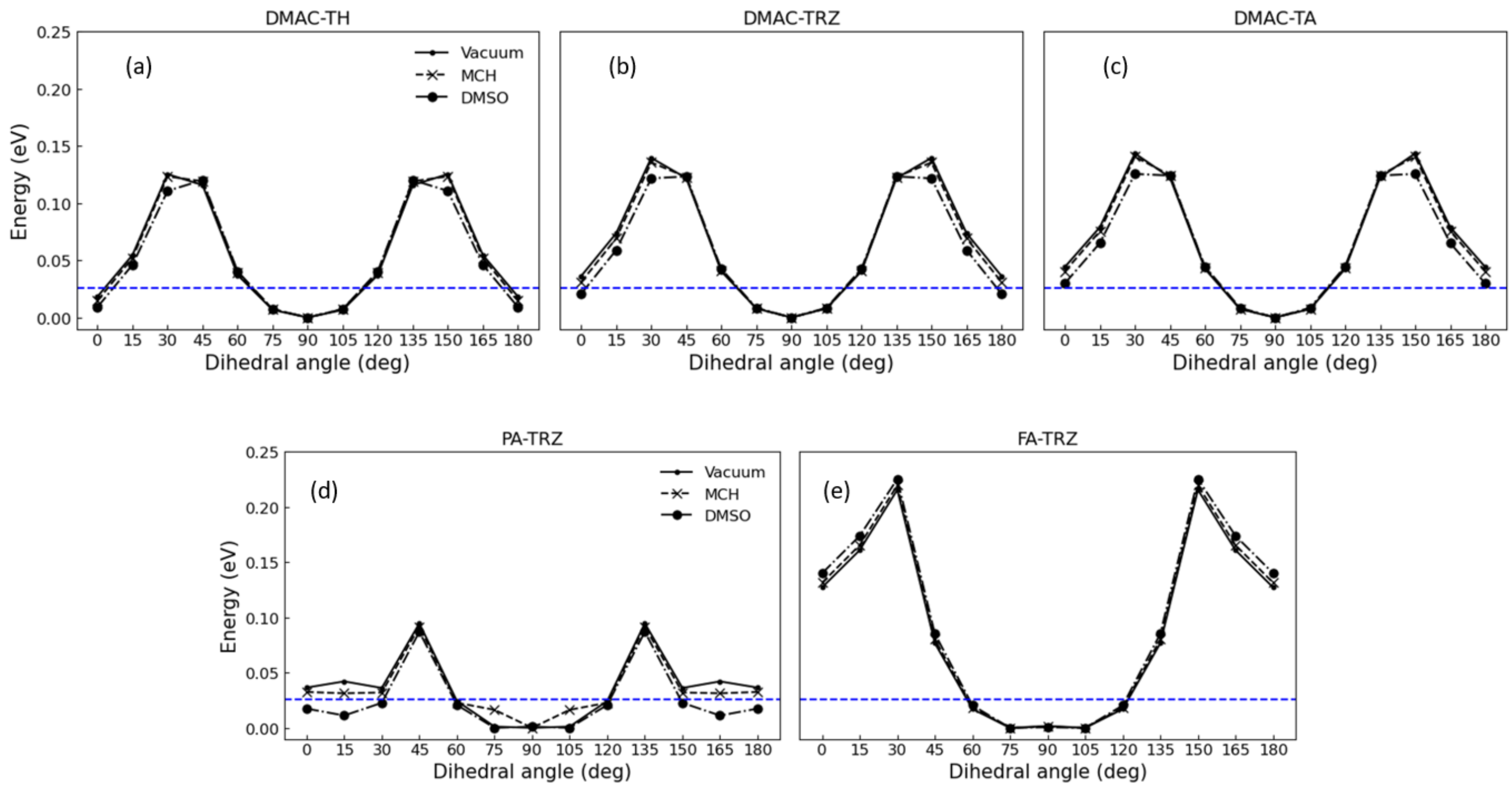

Figure S9 The relaxed energy scans of the ground state for the five molecules considered in the paper. The blue dashed line represents the thermal energy at room temperature.

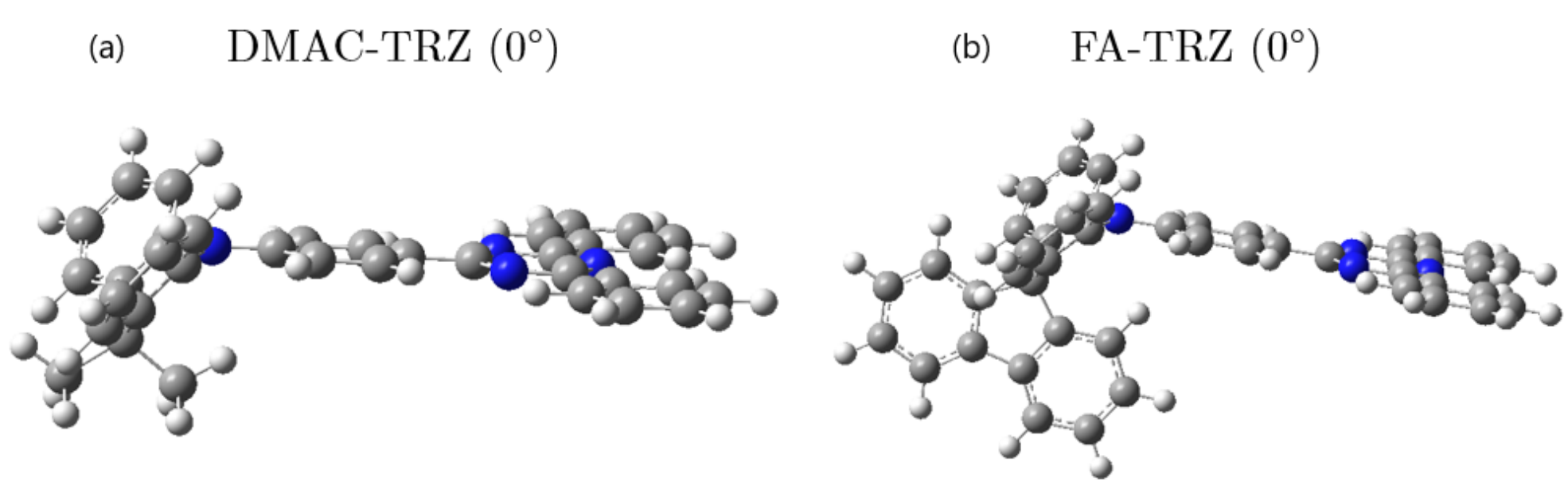

Figure S10 Quasi-axial conformations of DMAC-TRZ (a) and FA-TRZ b.

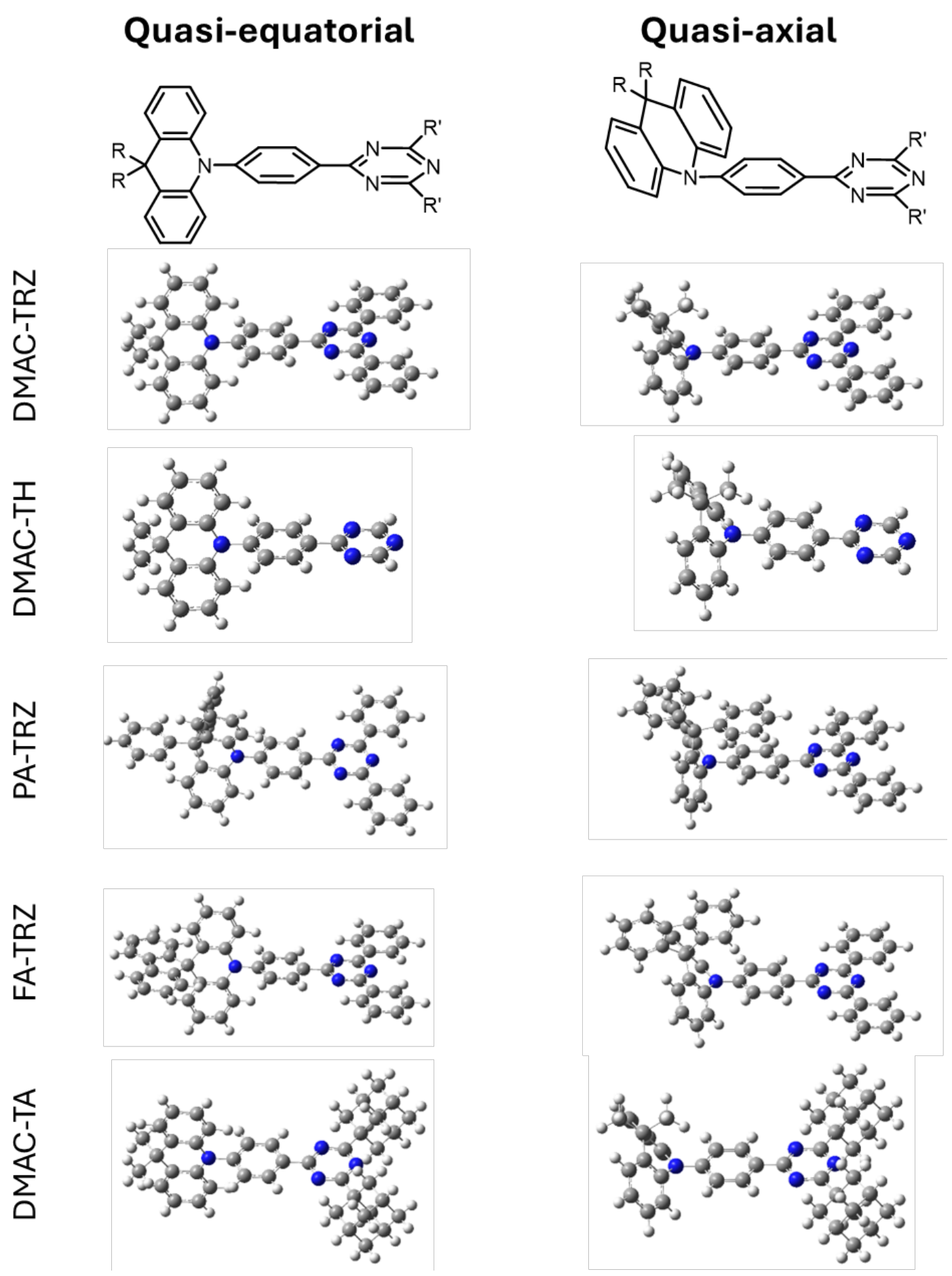

Figure S11 Sketch of the quasi-equatorial (left) and quasi-axial (right) conformations, and ground state quasi-equatorial and quasi-axial conformations for DMAC-TRZ, DMAC-TH, PA-TRZ, FA-TRZ, and DMAC-TA. The quasi-equatorial conformation corresponds to an approximately orthogonal donor-acceptor arrangement, while the quasi-axial conformation to a more folded, near-planar/periplanar arrangement.

4 Basis set dependence of excitation energies

Table S1 Gas phase excitation energies (in eV) for DMAC-TRZ for different levels of theory.

	T_1	S_1	T_2	T_3	T_4
6-31G(d)	3.44	3.45	3.78	3.83	3.97
6-31+G(d)	3.38	3.39	3.50	3.64	3.77
Def2TZVP	3.43	3.44	3.50	3.60	3.76

Table S2 Gas phase excitation energies (in eV) for DMAC-TH for different levels of theory.

	T_1	S_1	T_2	T_3	T_4
6-31G(d)	3.36	3.36	3.83	3.83	4.04
6-31+G(d)	3.29	3.30	3.60	3.64	3.87
Def2TZVP	3.35	3.36	3.60	3.61	3.83

Different basis sets affect mainly the energies of the local triplet states (T2, T3, T4), but, most importantly, the energy order is not affected, with the two CT states, S1 and T1, always lower in energy than the local triplet states. This trend is consistent with benchmarks, showing that TD-DFT excitation energies for low energy excited states of organic molecules are less affected by the choice of basis set than wavefunction methods (ref 47, main text).

5 Oscillator strengths

Table S3 Gas phase oscillator strengths of $S_0 \rightarrow S_1$ transition for the 5 molecules of interest at their ground state geometry.

Molecule	f_{osc}
FA-TRZ	$1.1 \cdot 10^{-3}$
PA-TRZ	$6 \cdot 10^{-4}$
DMAC-TRZ	$< 10^{-4}$
DMAC-TA	$< 10^{-4}$
DMAC-TH	$< 10^{-4}$

6 Spin orbit couplings

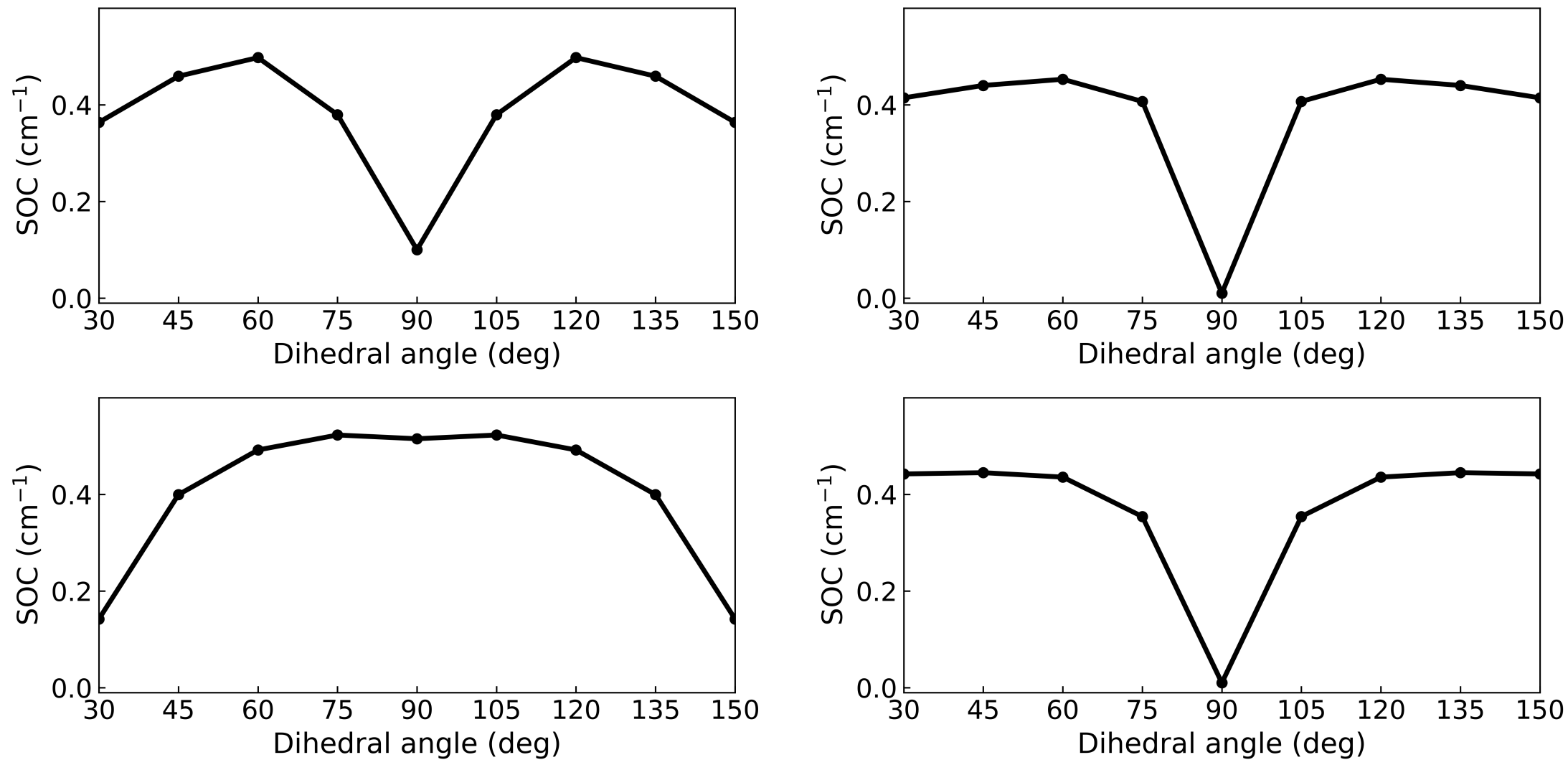

Figure S12 The $S_1 - T_1$ SOC computed at the geometries obtained relaxing the ground state geometries of FA-TRZ (upper left panel), DMAC-TA (upper right panel), PA-TRZ (lower left panel) and DMAC-TH (lower right panel).

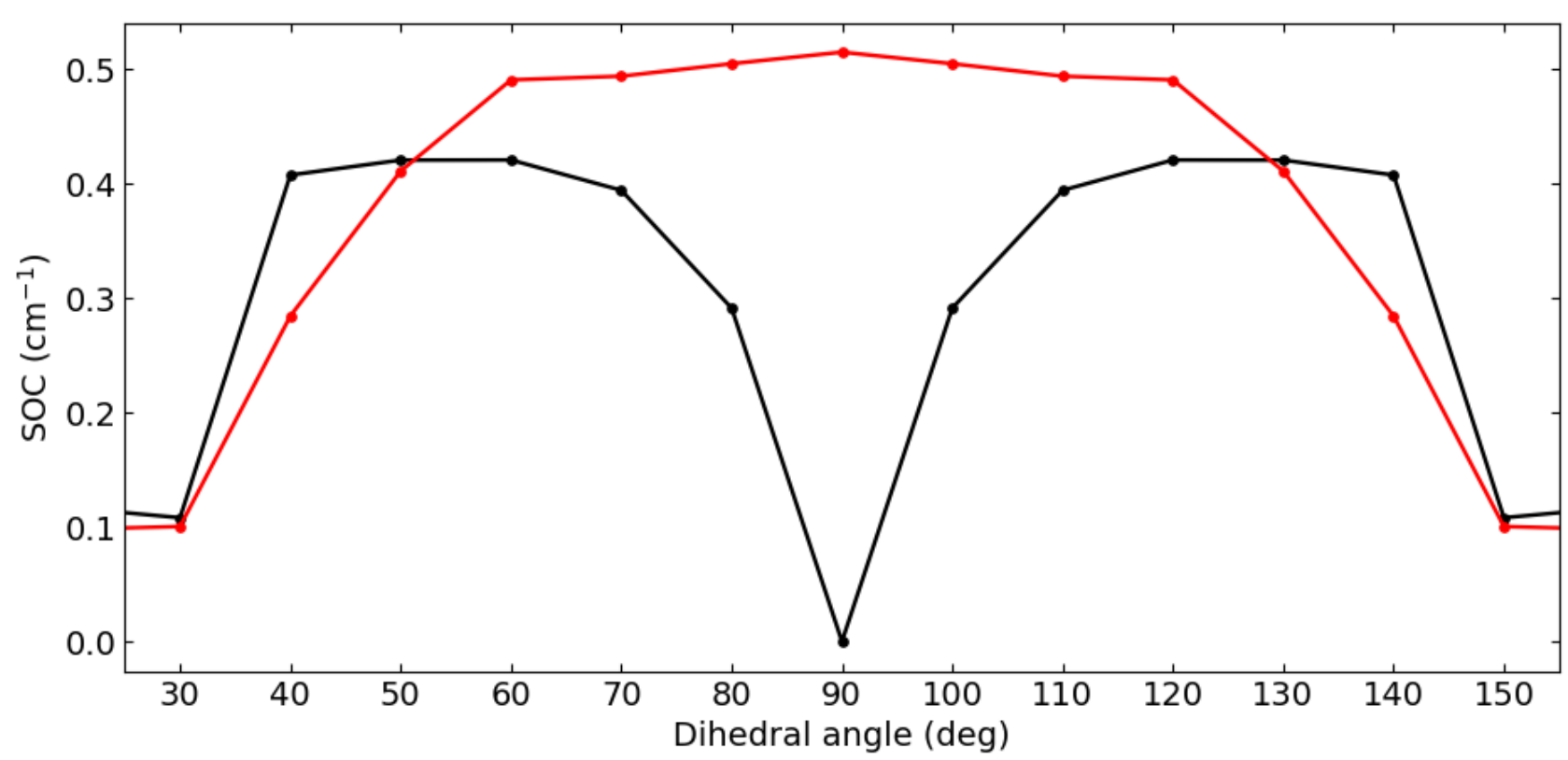

Figure S13 Comparison of the $S_1 - T_1$ SOC of PA-TRZ (red) and DMAC-TRZ (black) as a function of the dihedral angle (relaxed scan).

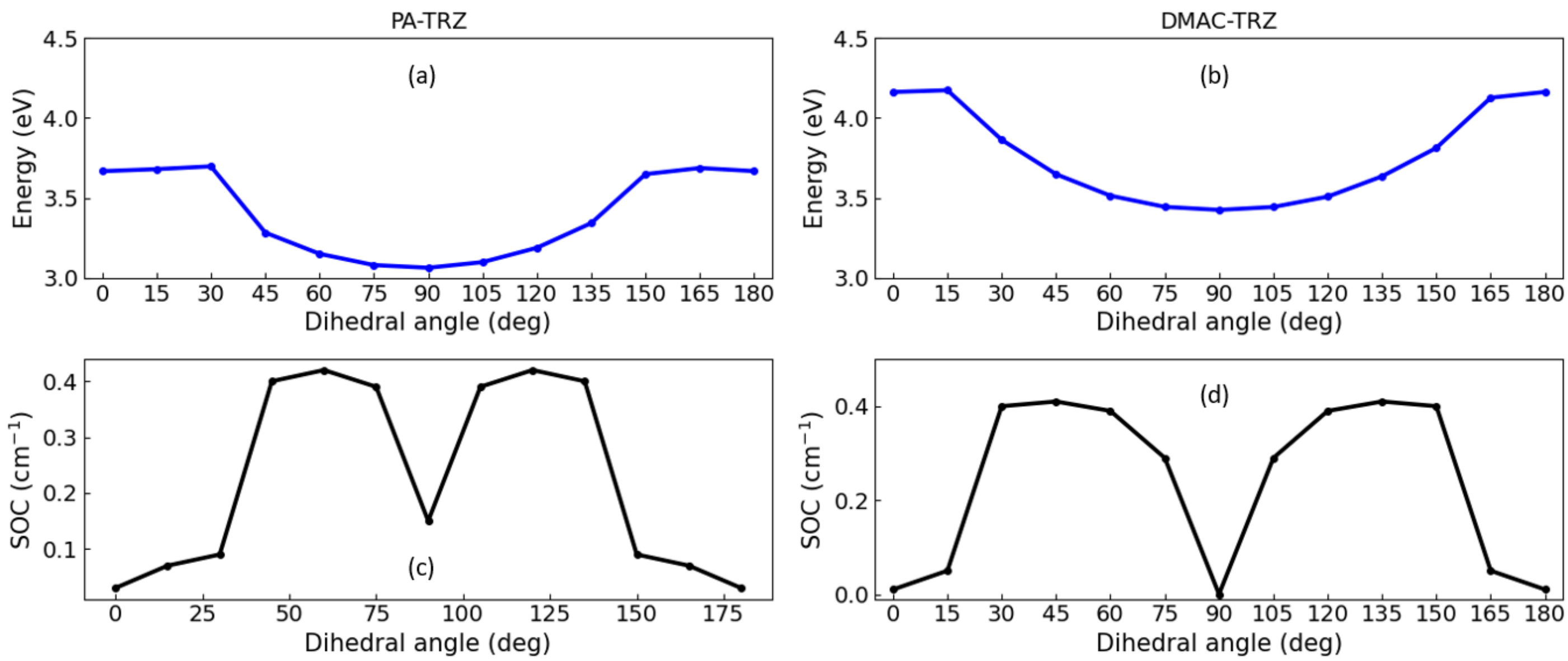

Figure S14 (a)Top panels: the energies of the S_1 state (blue) calculated for PA-TRZ (l) and DMAC-TRZ (right panels) as a function of the dihedral angle for the S_1 optimized geometry (relaxed scan). Bottom panels: the corresponding SOC matrix element between S_1 and T_1.

7 Analysis of the fluorescence decay

The total intensity of the emitted light as a function of the time $I(t)$ is analyzed to extract the rates of relevant photophical processes. The first step in the process is the fit of the decay curves (in Fig. S7 and S8) with a double exponential function:

$$I(t) = A_p \exp(-k_p t) + A_d \exp(-k_d t) \tag{S1}$$

where A_p and A_d are the pre-exponential factors (data is normalized at $t = 0$ so that $A_p + A_d = 1$), k_p and k_d are the rates of prompt and delayed emission, respectively. The experimental data and the fitting curves are shown in Figs 7 and 8 for DMAC-TRZ and PA-TRZ, respectively. The relevant fit parameters are listed in Table 1.

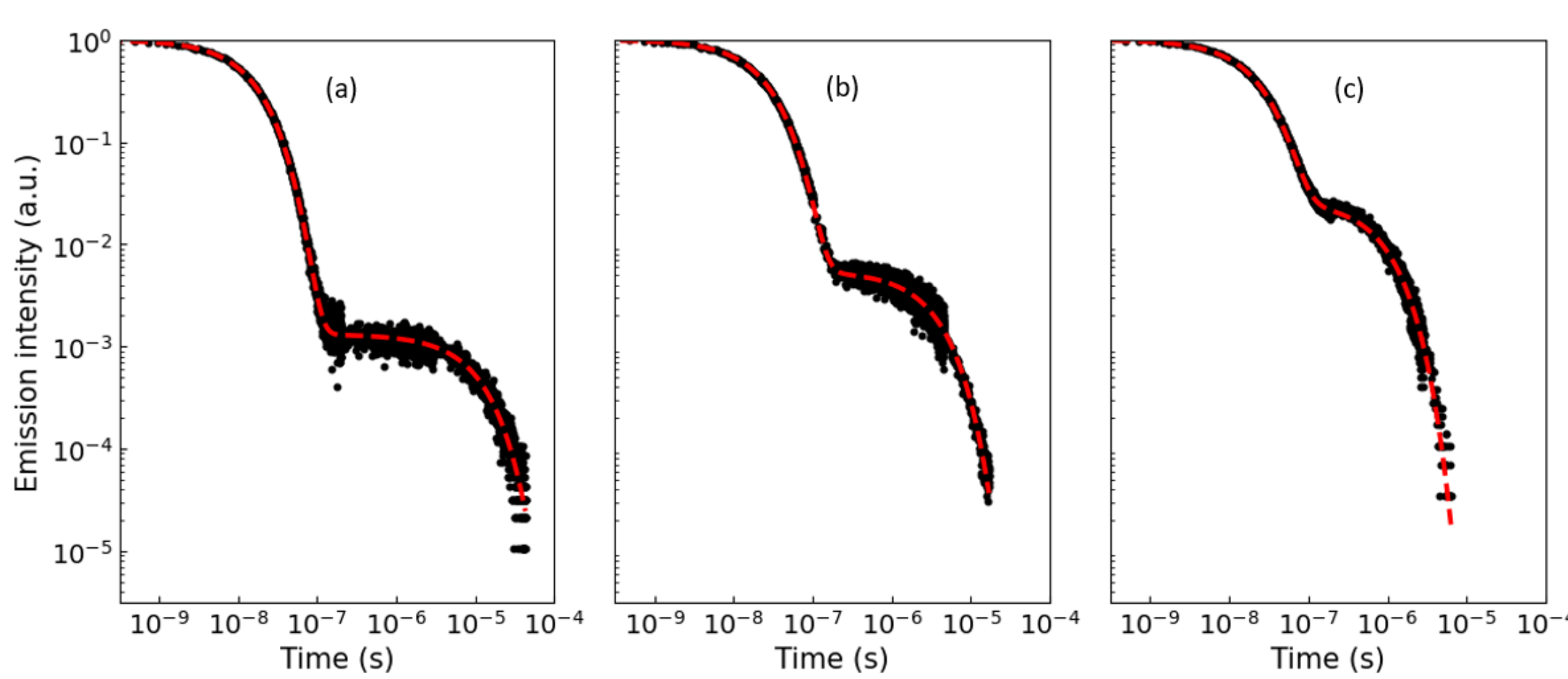

Figure S15 Fitting of the emission intensity as a function of time for DMAC-TRZ in cyclohexane (left panel), toluene (central panel) and chloroform (right panel). Black dots and red dashed lines represent the experimental points and the fitted curve, respectively.

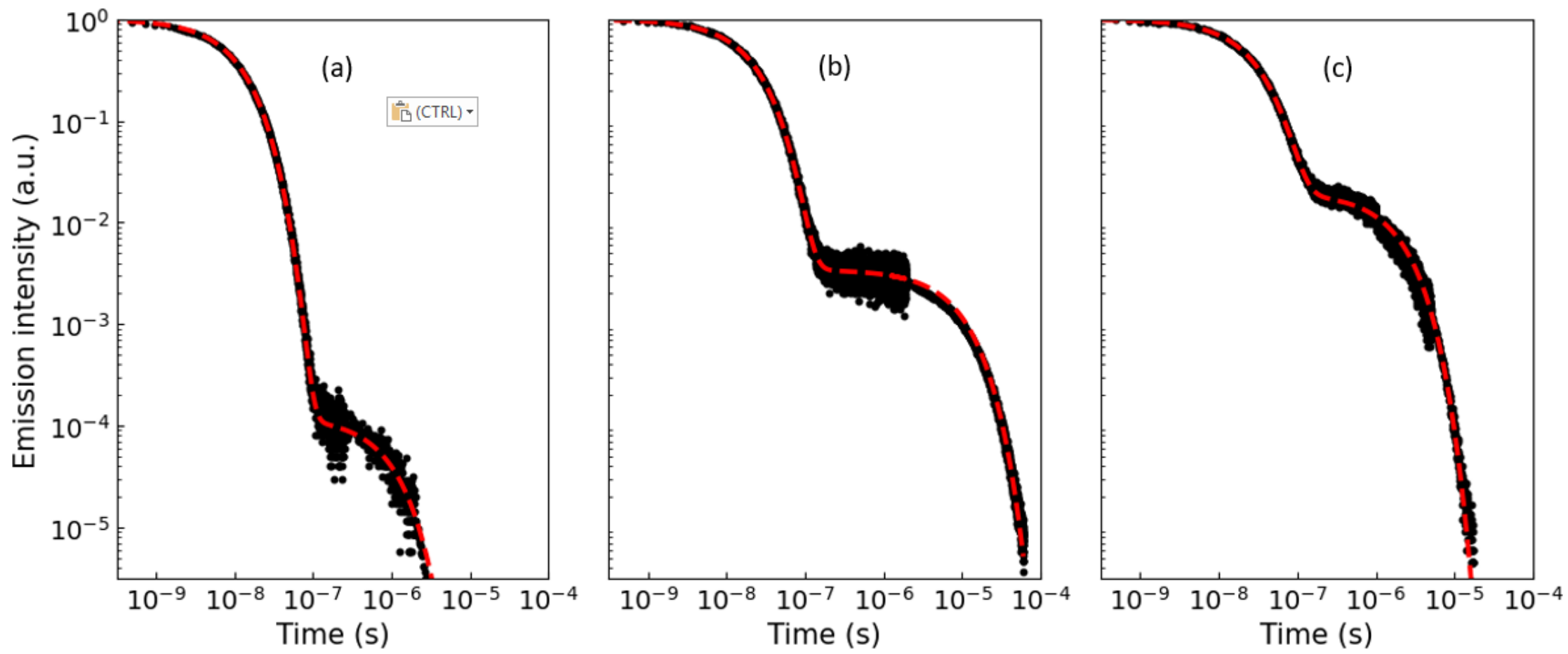

Figure S16 Fitting of the emission intensity as a function of time for PA-TRZ in cyclohexane (left panel), toluene (central panel) and chloroform (right panel). Black dots and red dashed lines represent the experimental points and the fitted curve, respectively.

Table S4 Results of the double exponential fitting for DMAC-TRZ and PA-TRZ in different solvents.

	**DMAC-TRZ**			**PA-TRZ**		
	Cyclohexane	Toluene	Chloroform	Cyclohexane	Toluene	Chloroform
$A_p(\%)$	99.9	99.5	97.3	99.99	99.7	98.0
$A_d(\%)$	0.1	0.05	2.7	0.01	0.3	2.0
$k_p(\mathrm{s}^{-1})$	$6.28 \cdot 10^7$	$3.90 \cdot 10^7$	$4.45 \cdot 10^7$	$9.20 \cdot 10^7$	$4.72 \cdot 10^7$	$3.62 \cdot 10^7$
$k_d(\mathrm{s}^{-1})$	$9.29 \cdot 10^4$	$2.96 \cdot 10^5$	$1.15 \cdot 10^6$	$1.12 \cdot 10^6$	$1.08 \cdot 10^5$	$5.36 \cdot 10^5$

Adopting the theoretical model and the approximations reported in ref.[4], data in Table S4 allow us to extract the rates of the decay processes of the two molecules in liquid environment as reported in Tables S5 and S6.

Table S5 Experimental rate constants for DMAC-TRZ in different solvents. For the rISC and ISC rates, the maximum and minimum values set the relevant error bars.

	Cyclohexane	Toluene	Chloroform
$\mathrm{k_r^S}$	$7.3 \cdot 10^6$	$4.1 \cdot 10^6$	$5.1 \cdot 10^6$
$\mathrm{k_{nr}^S}$	$2.6 \cdot 10^7$	$1.9 \cdot 10^7$	$1.7 \cdot 10^7$
$\mathrm{k_{nr}^T}$	$8.2 \cdot 10^4$	$2.7 \cdot 10^5$	$1.0 \cdot 10^6$
$\mathrm{k_{ISC,ave}}$	$4.2 \cdot 10^7$	$2.6 \cdot 10^7$	$3.1 \cdot 10^7$
$\mathrm{k_{ISC,max}}$	$5.5 \cdot 10^7$	$3.5 \cdot 10^7$	$3.9 \cdot 10^7$
$\mathrm{k_{ISC,min}}$	$3.0 \cdot 10^7$	$1.6 \cdot 10^7$	$2.2 \cdot 10^7$
$\mathrm{k_{rISC,ave}}$	$1.3 \cdot 10^5$	$3.7 \cdot 10^5$	$1.8 \cdot 10^6$
$\mathrm{k_{rISC,max}}$	$1.8 \cdot 10^5$	$5.1 \cdot 10^5$	$2.3 \cdot 10^6$
$\mathrm{k_{rISC,min}}$	$9.4 \cdot 10^4$	$2.4 \cdot 10^5$	$1.3 \cdot 10^6$

Table S6 Experimental rate constants for PA-TRZ in different solvents. For the rISC and ISC rates, the maximum and minimum values set the relevant error bars. Please notice the very large uncertainty for the RISC and ISC rates collected in cyclohexane, as also shown in Fig. 7

	Cyclohexane	Toluene	Chloroform
k_r^S	$1.8 \cdot 10^7$	$4.0 \cdot 10^6$	$2.7 \cdot 10^6$
k_{nr}^S	$7.3 \cdot 10^7$	$1.5 \cdot 10^7$	$1.3 \cdot 10^7$
k_{nr}^T	$1.1 \cdot 10^6$	$9.4 \cdot 10^4$	$4.8 \cdot 10^5$
$k_{ISC,ave}$	$3.7 \cdot 10^7$	$3.6 \cdot 10^7$	$2.7 \cdot 10^7$
$k_{ISC,max}$	$4.7 \cdot 10^7$	$4.3 \cdot 10^7$	$3.4 \cdot 10^7$
$k_{ISC,min}$	$8.9 \cdot 10^5$	$2.8 \cdot 10^7$	$2.0 \cdot 10^7$
$k_{rISC,ave}$	$5.7 \cdot 10^5$	$2.2 \cdot 10^5$	$9.9 \cdot 10^5$
$k_{rISC,max}$	$1.1 \cdot 10^6$	$2.7 \cdot 10^5$	$1.2 \cdot 10^6$
$k_{rISC,min}$	$1.4 \cdot 10^4$	$1.8 \cdot 10^5$	$7.5 \cdot 10^5$

8 Essential state analysis applied to PA-TRZ

We adapt the semi-empirical model that was successfully applied to DMAC-TRZ to PA-TRZ. The model relies on two singlet basis states, N and Z, corresponding to a neutral and a zwitterionic structure, separated by an energy gap $2z$ and coupled by a hopping term τ. The triplet manifold consists of a zwitterionic triplet, T, degenerate with the zwitterionic singlet, and a single effective local state, L, lying at energy $2k$ above the neutral state and coupled to T via a matrix element β. Spin-orbit coupling is introduced following the El-Sayed rule, *i.e.* directly coupling only states with different spin and electronic nature. The dihedral angle between the donor and acceptor units controls the amount of conjugation between the two moieties, effectively modulating the couplings τ and β. The Hamiltonian adopted for PA-TRZ is then the following (basis: N, Z, T, L):

$$\mathcal{H}_{el} = \begin{pmatrix} 0 & -\tau_1|\sin\delta| & V_{soc} & 0 \\ -\tau_1|\sin\delta| & 2z & 0 & W_{soc} \\ V_{soc} & 0 & 2z & -\beta|\sin\delta| \\ 0 & W_{soc} & -\beta|\sin\delta| & 2k \end{pmatrix} + a_2\delta^2 + a_4\delta^4 \tag{S2}$$

where a_2 and a_4 are the prefactors for the quadratic and quartic terms of the restoring potential along the dihedral, and $\delta = \theta - 90°$, θ being the dihedral angle between donor and acceptor. The parameters entering eq. S2 are extracted from *ab initio* calculations, fitting the PES of the lowest excited states of PA-TRZ and the dependence of SOC vs the dihedral angle (Fig. S17). Here, in order to better compare PA-TRZ and DMAC-TRZ, we decided to modify as few parameters required to get a satisfactory qualitative agreement between model and *ab initio*.

The main difference between the model in eq. S2 for PA-TRZ and the one in ref.[5] for DMAC-TRZ lies in the dependence of the diabatic coupling between triplets: in order to properly reproduce the SOC dependence and PES obtained ab initio (Fig. S17), a constant term β_0 has to be introduced. The energy and SOC dependence on θ for DMAC-TRZ and PA-TRZ is shown in Fig. S17, using parameters from Table S7.

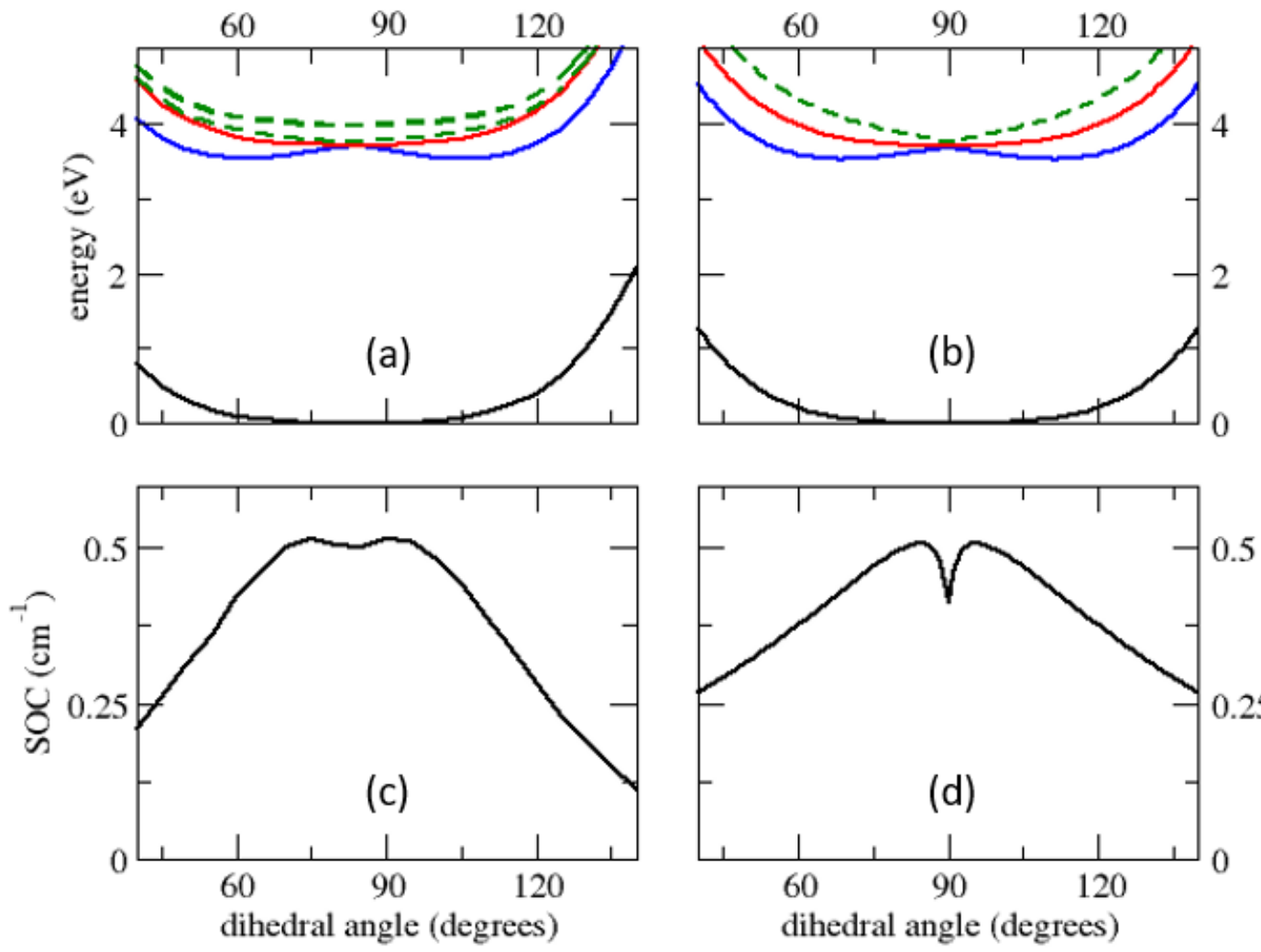

Figure S17 Left panels show ab initio results for PA-TRZ: PES of the lowest excited states (top) and $S_1 - T_1$ SOC (bottom). Right panels show simulated results obtained with the adopted few-state model for PA-TRZ: PES of the lowest excited states (top) and $S_1 - T_1$ SOC (bottom) of PA-TRZ, with parameters in Table S7.

Table S7 Model parameters for DMAC-TRZ and PA-TRZ

	DMAC-TRZ	PA-TRZ
z (eV)	1.72	1.85
τ_1 (eV)	0.75	0.75
k (eV)	1.96	1.96
β	0.85	0.85
a_2	$6.00 \cdot 10^{-5}$	$6.00 \cdot 10^{-5}$
a_4	$1.43 \cdot 10^{-7}$	$1.43 \cdot 10^{-7}$
V_{soc}	$3.84 \cdot 10^{-4}$	$3.84 \cdot 10^{-4}$
W_{soc}	$1.74 \cdot 10^{-4}$	$1.74 \cdot 10^{-4}$

In order to address experimental spectra, two other ingredients are required, namely, an effective molecular vibration that leads to vibronic progression and solvation. We introduce a molecular mode, $\hat{Q}$ (momentum $\hat{P}$), with frequency $\hbar\omega_v = 0.18$ eV, which is coupled to the zwitterionic states and accounts for the molecular relaxation following charge transfer, with relaxation energy ϵ_v= 0.17 eV. Finally, solvation is taken into account in the reaction-field model, where the elastic medium responds to the presence of a solute molecule generating at the solute location an electric field proportional to the solute dipole moment. The reaction field is partitioned into an electronic reaction field, F_{el}, treated in the antiadiabatic approximation, and an orientational field, F_{or}, treated in the adiabatic approximation. The antiadiabatic approximation in F_{el} leads to a renormalization of the energy of the zwitterionic states by an amount that corresponds to the electronic solvent reorganization energy, ϵ_{el}, which depends on the molecule and on the refractive index of the medium. We set this value to ϵ_{el} = 0.55 eV for all solvents of interest due to the low variability of refractive indexes of organic solvents, and to best reproduce the position of the absorption bands (Fig. S18).

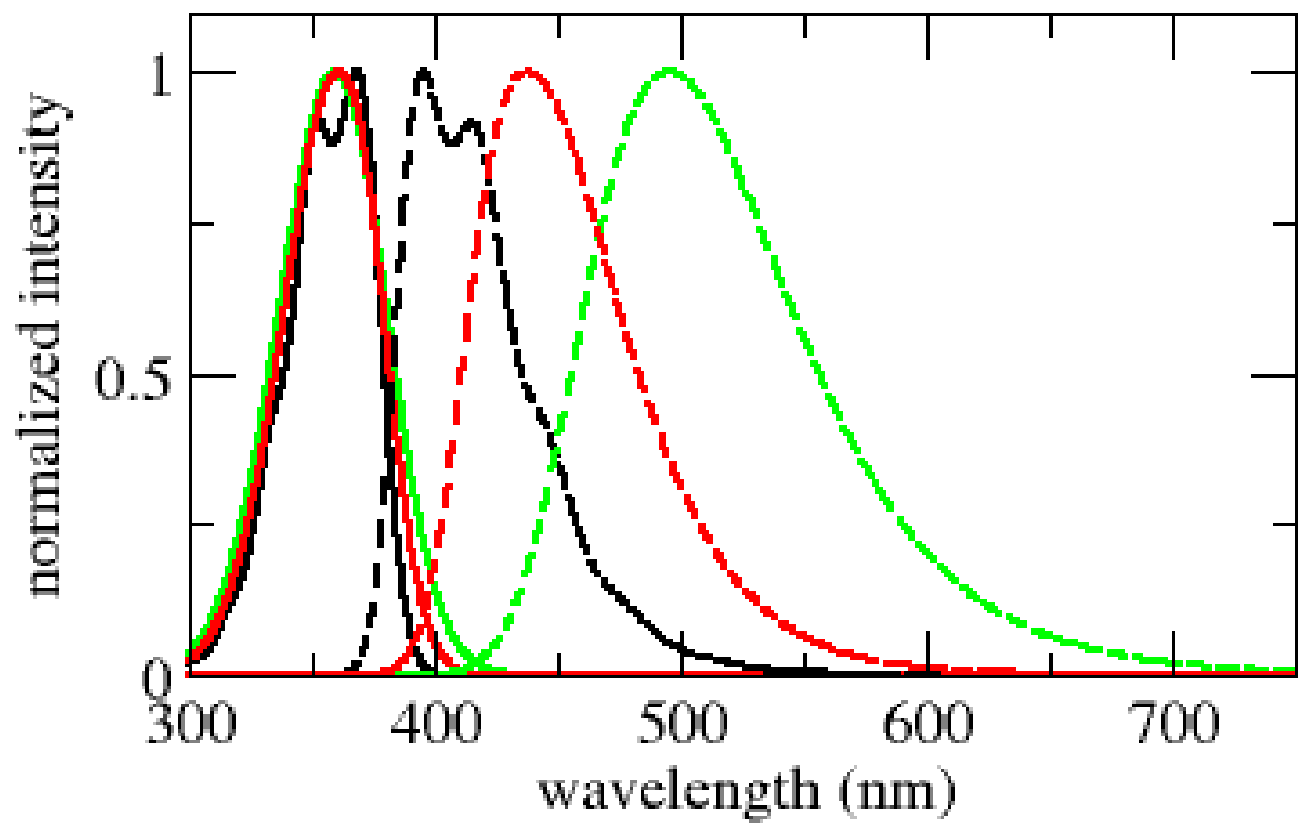

Figure S18 Normalized absorption (continuous lines) and fluorescence spectra (dashed lines) calculated for PA-TRZ in cyclohexane, toluene and chloroform. The solvent parameters are set to $\epsilon_{el} = 0.45\,\mathrm{eV}$ (for all solvents) and $\epsilon_{or} = [0.12, 0.22, 0.38]$ eV for cyclohexane, toluene, and chloroform respectively. The HWHM associated to each transition is $\Gamma = 0.03\,\mathrm{eV}$.

The orientational component of the solvent reaction field, F_{or}, is treated as a classic coordinate, leading to the following Hamiltonian

$$\mathcal{H} = \mathcal{H}'_{el} + \left[-\sqrt{\hbar\omega_v\epsilon_v}\hat{Q}\left(|Z\rangle\langle Z| + |T\rangle\langle T|\right) - \sqrt{\hbar\omega_v\epsilon_v^{(L)}}\hat{Q}\,|L\rangle\langle L| + \frac{\hbar\omega_v}{4}\left(\hat{Q}^2 + \hat{P}^2\right)\right] + \left[-F_{or}\left(|Z\rangle\langle Z| + |T\rangle\langle T|\right) + \frac{1}{4\epsilon_{or}}F_{or}^2\right] \quad \text{(S3)}$$

where $\mathcal{H}'_{el}$ is the Hamiltonian in eq. S2, renormalized, as discussed above to account for the electronic component of the solvation field. The second term in (S3) accounts for additional vibrational relaxation of the local triplet state, with relaxation energy $\epsilon_v^{(L)} = 0.145\,\mathrm{eV}$. Moreover, ϵ_{or} is the orientational solvent relaxation energy, a quantity that increases with the solvent polarity. The parameters for the effective vibrational mode $\hat{Q}$ of PA-TRZ are set equal to those of DMAC-TRZ, while ϵ_{or} values are slightly adjusted to best reproduce the solvatochromism of PA-TRZ spectra (Fig. S18).

In order to address ISC and RISC rates, k_{ISC} and k_{RISC}, we adopt the non-equilibrium Fermi's golden rule approach proposed in ref.[5]. Specifically, we solve the total Hamiltonian (parametric in F_{or}) accounting for $\hat{\delta}$ as a quantum coordinate in second quantization, and for SOC as a perturbation. Each state is assigned a finite lifetime $\tau = 100\,\mathrm{fs}$, so that the relevant energy is broadened by a Gaussian lineshape with $\sigma = \frac{1}{2\pi\sqrt{2\ln 2}\tau_r}$. The state-to-state transition rates are finally calculated, averaging over the thermal population of the initial state (either the singlet, for ISC, or the triplet, for RISC). Along these lines, ISC and RISC rates are obtained that depend on F_{or}, as shown in Fig. S19. The rates relevant to each solution are finally obtained by averaging over the relevant F_{or}-distribution. Table IV in the main text collects the resulting ISC and RISC rates in solution for PA-TRZ and DMAC-TRZ (from ref.[6]).

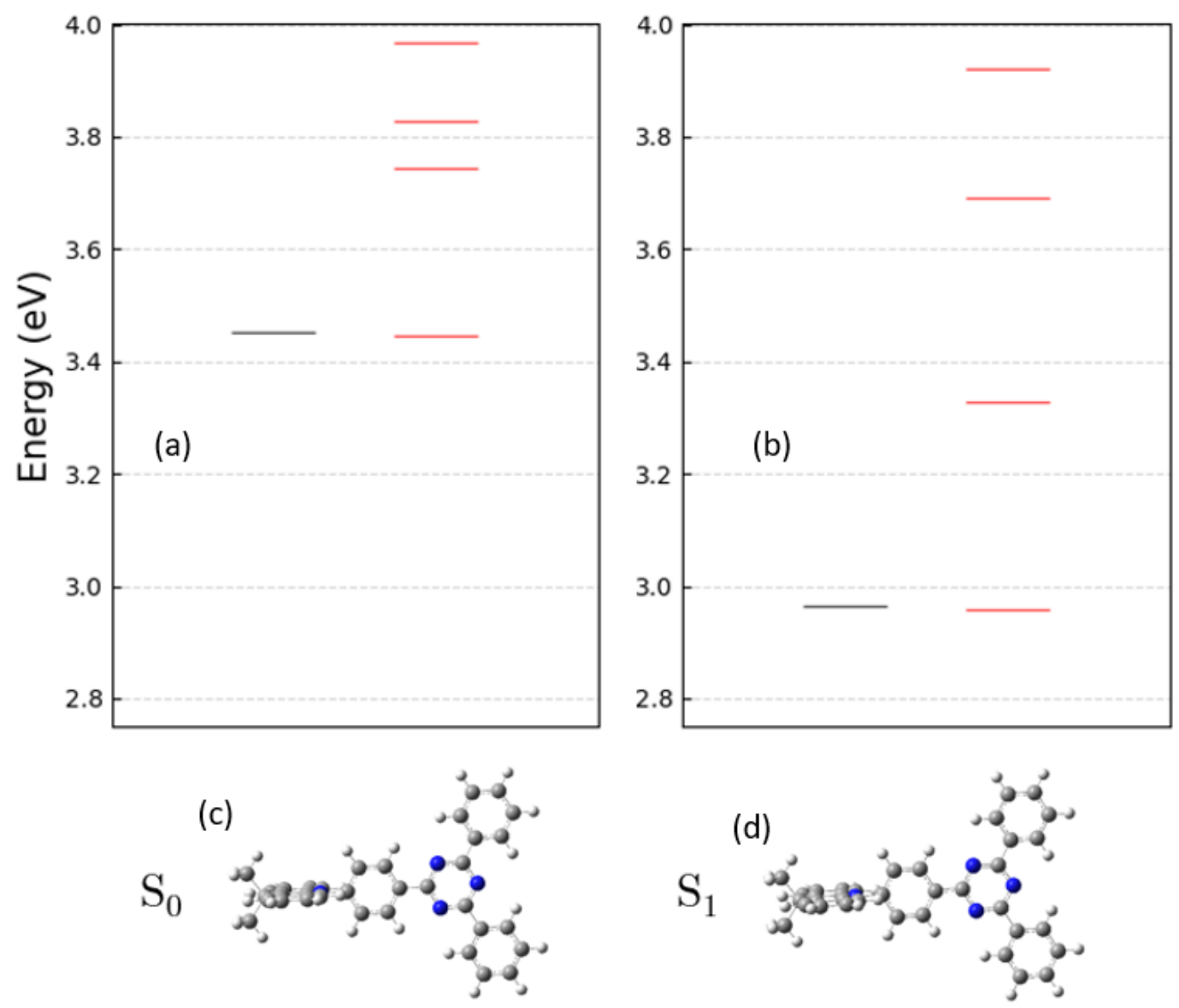

Figure S20 Top panels: The absolute energies of S_1 (black) and of the first few triplet states (red) calculated for DMAC-TRZ at the S_0 and S_1 equilibrium geometries (left and right panel, respectively). Bottom panel, the corresponding geometries.

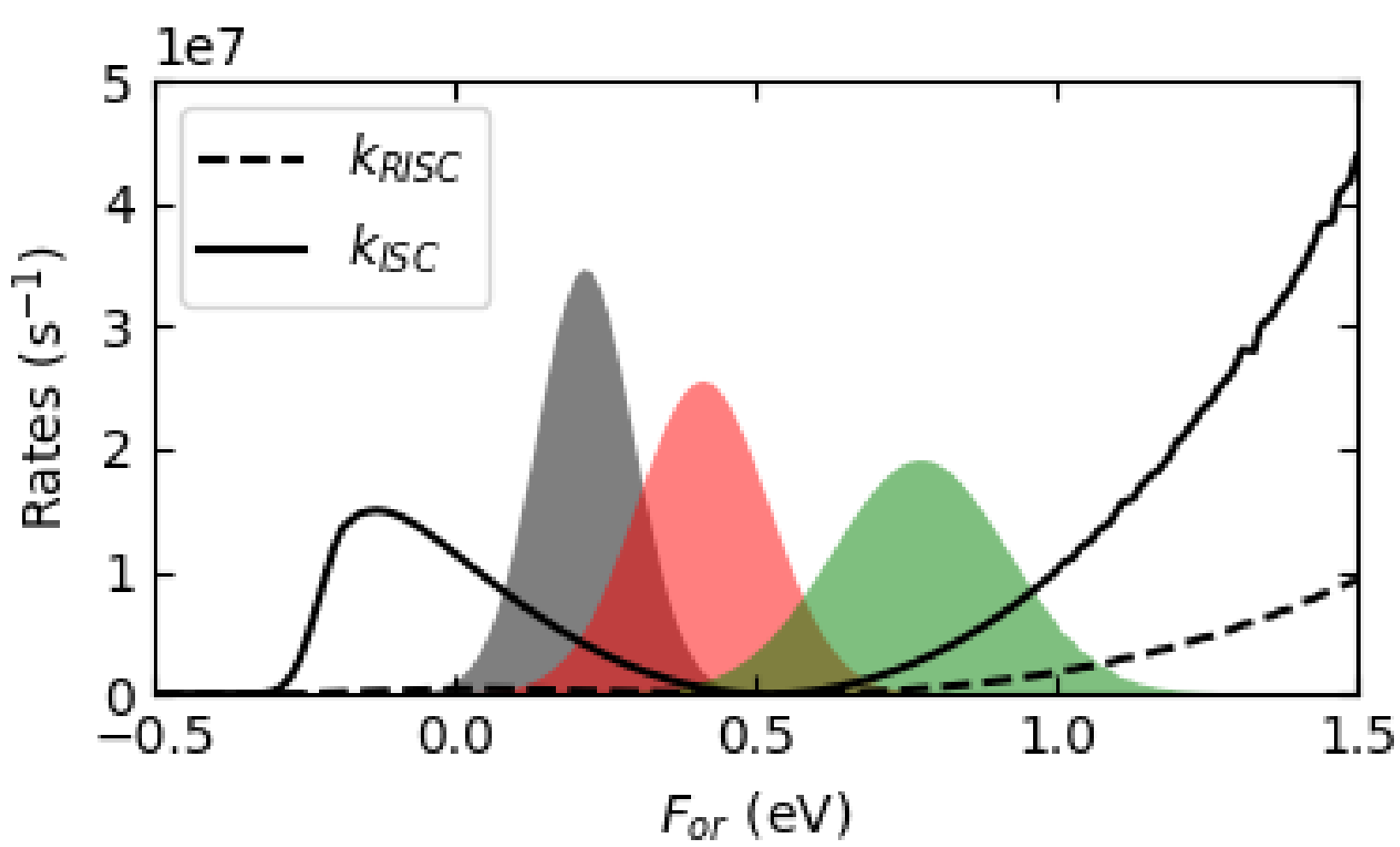

Figure S19 ISC (continuous) and RISC rates (dashed) for PATRZ as a function of F_{or}. The shaded areas represent the triplet distributions for cyclohexane (black), toluene (red) and chloroform (green).

Notes and references

[1] T. Lin, T. Chatterjee, W. Tsai, W. Lee, M. Wu, M. Jiao, K. Pan, C. Yi, C. Chung, K. Wong and C. Wu, *Advanced Materials*, 2016, **28**, 6976–6983.

[2] Y. Wada, S. Kubo and H. Kaji, *Adv. Mater.*, 2018, **30**, 1705641.

[3] B. L. Buss, C.-H. Lim and G. M. Miyake, *Angew. Chem., Int. Ed.*, 2020, **59**, 3209–3217.

[4] Y. Tsuchiya, S. Diesing, F. Bencheikh, Y. Wada, P. L. dos Santos, H. Kaji, E. Zysman-Colman, I. D. W. Samuel and C. Adachi, *The Journal of Physical Chemistry A*, 2021, **125**, 8074–8089.

[5] D. K. A. P. Huu, S. Saseendran and A. Painelli, *J. Mater. Chem. C*, 2022, **10**, 4620–4628.

[6] D. K. A. P. Huu, S. Saseendran, R. Dhali, L. G. Franca, K. Stavrou, A. Monkman and A. Painelli, *Journal of the American Chemical Society*, 2022, **144**, 15211–15222.
